# Engineering BiTE-inspired IPSC-exosomes to potentiate CAR-T cell therapy against lung cancer

**DOI:** 10.1186/s12951-026-04242-3

**Published:** 2026-03-17

**Authors:** Ronghao Wang, Guining Fu, Haozhao Dou, Mingyuan Hu, Zhanglin Li, Junwen Gan, Jiasheng He, Xiaojian Li, Guihong Zhang, Xianjun Li, Tianchuan Zhu, Qingdong Cao

**Affiliations:** 1 Department of Thoracic Surgery, The Fifth Affiliated Hospital of Sun Yat-sen University, Zhuhai, 519000 Guangdong China; 2 Center for Infection and Immunity, Guangdong Engineering Research Center of Molecular Imaging, The Fifth Affiliated Hospital of Sun Yat-sen University, Zhuhai, 519000 Guangdong China; 3 Department of Breast Surgery, Harbin Medical University Cancer Hospital, Harbin, 150081 Heilongjiang China

**Keywords:** Chimeric antigen receptor T cells, Induced pluripotent stem cells, Exosomes, Bispecific T cell engagers, Lung cancer, Immunotherapy, Indole-3-propionic acid

## Abstract

Chimeric antigen receptor T (CAR-T) cell therapy faces critical barriers in solid tumors, including poor infiltration, T cell exhaustion, and immunosuppressive microenvironments, resulting in response rates below 10%. Herein, we engineered an inhalable nanoplatform using induced pluripotent stem cell-derived exosomes (IEXOs) displaying bispecific PD-1/mesothelin (MSLN) single-chain variable fragments (scFv) and loaded with indole-3-propionic acid (IPA) for metabolic reprogramming. IEXOs demonstrated high yield and intrinsic antitumor properties, inhibiting Lewis lung carcinoma (LLC) cell proliferation and migration. The bispecific exosomes loaded with IPA (BIEXO@IPA) achieved efficient pulmonary delivery *via* nebulization with 79.3% tumor cell-specific uptake versus 47.9% for liposomes in orthotopic lung cancer models. BIEXO@IPA treatment reduced tumor burden by 87.9% and achieved 80% survival at 80 days. Mechanistically, BIEXO@IPA bridged PD-1^+^ T cells to MSLN^+^ tumor cells through bispecific engagement while expanding progenitor exhausted T (Tpex) cells and reducing regulatory T cells. When combined with CAR-T cells, BIEXO@IPA achieved 66.7% complete remission with 100% survival at 80 days and 83.3% resistance to tumor rechallenge. Safety assessments revealed minimal toxicity. This BIEXO@IPA platform represents a scalable, clinically translatable strategy that addresses fundamental CAR-T limitations in solid tumors through synergistic multimodal immunomodulation.

**Graphical Abstract:**

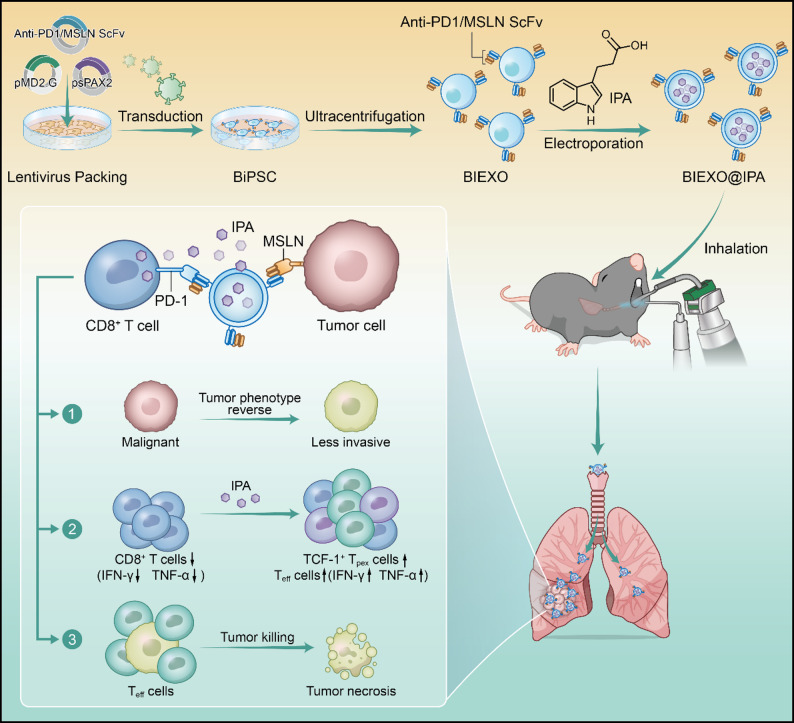

**Supplementary Information:**

The online version contains supplementary material available at 10.1186/s12951-026-04242-3.

## Introduction

Chimeric antigen receptor T-cell (CAR-T) therapy has revolutionized cancer treatment, achieving complete remission rates of 80–90% in B-cell acute lymphoblastic leukemia and 40–70% in refractory multiple myeloma [1–4]. Six FDA-approved CAR-T products have established this modality as a cornerstone of hematologic cancer therapy [1, 5]. However, this success starkly contrasts with solid tumor outcomes, where pooled response rates remain at merely 9% across 262 patients in multiple clinical trials [6]. This disparity is particularly evident in lung cancer, where despite 69% of adenocarcinomas expressing targetable antigens like mesothelin (MSLN), CAR-T approaches demonstrate minimal efficacy with frequent disease progression [7–10]. The fundamental disconnect between liquid and solid tumor outcomes represents one of the most pressing challenges in contemporary oncology.

The biological barriers limiting CAR-T efficacy in solid tumors are multifaceted and synergistic. T cell exhaustion, marked by inhibitory receptor upregulation and effector function loss, represents a dominant barrier [11, 12]. Additionally, < 5% of CAR-T cells reach tumors due to physical barriers [1]. The immunosuppressive tumor microenvironment (TME) further compounds these challenges through myeloid-derived suppressor cells, regulatory T cells (Tregs), and soluble factors including TGF-β, adenosine, and kynurenine, creating a profoundly inhibitory milieu that rapidly renders CAR-T cells dysfunctional [1, 13, 14]. 

Current combination strategies have shown promise but face significant limitations. Checkpoint blockade combined with MSLN-CAR-T achieved 72% response rates in mesothelioma [15]. However, CRISPR-mediated PD-1 disruption unexpectedly impaired CAR-T persistence in clinical trials, while shRNA-mediated knockdown preserving partial PD-1 function yielded superior outcomes with 85.7% response rates in myeloma [16]. Bispecific T-cell engager (BiTE) combinations address antigen heterogeneity, with CAR-T cells secreting BiTEs demonstrating enhanced sensitivity to low-antigen tumors and 3-fold increased cytotoxicity in lymphoma models [17, 18]. Yet BiTEs face practical challenges including rapid clearance (2–3 h half-life), potential Treg recruitment, and manufacturing complexity that delays treatment initiation [19–23]. 

Recent discoveries provide opportunities for innovation. TCF-1⁺PD-1⁺CD8⁺ progenitor exhausted T cells (Tpex) maintain self-renewal capacity during chronic antigen exposure and represent primary responders to checkpoint blockade [24, 25]. While indole-3-propionic acid (IPA) enhances Tpex formation and PD-1 therapy efficacy through metabolic reprogramming in preclinical models, its application in CAR-T therapy remains unexplored [26, 27]. Concurrently, exosome-based delivery platforms offer extended circulation through CD47-mediated immune evasion, with engineered approaches achieving efficient antibody display and superior tumor penetration [28, 29]. Conventional exosome sources, such as T cells, mesenchymal stem cells, and tumor cells are constrained by low yields, whereas embryonic stem cell (ESC)-derived exosomes display high yield of exosome and intrinsic antitumor effects but face ethical and sourcing limitations [30–32]. Addressing these, we turned to induced pluripotent stem cells (iPSCs), which mirror ESCs in function while enabling scalable production and genetic engineering [32–35]. Notably, our investigations reveal that iPSC-derived exosomes display high yield of exosome and intrinsically suppress tumor cell malignancy, inhibiting proliferation and invasion.

**Scheme 1:**
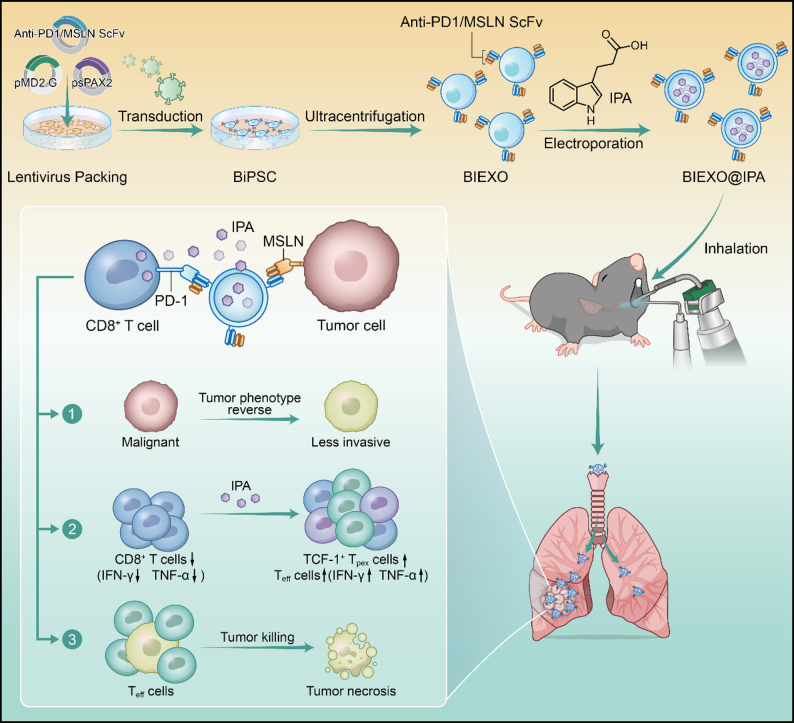
BIEXO@IPA platform for enhanced CAR-T therapy in lung cancer. In this study, iPSCs were lentivirally transduced with anti-PD-1/MSLN scFv to generate iPSC lines expressing the engineered construct (BiPSC). BIEXOs were isolated and concentrated from BiPSC culture supernatant *via* ultracentrifugation, then loaded with IPA through electroporation to form BIEXO@IPA. This platform was nebulized into orthotopic lung cancer mouse models for targeted delivery. In the lung TME, BIEXO@IPA bridges endogenous PD-1^+^ CD8^+^ T/CAR-T cells to MSLN-expressing tumor cells while blocking PD-1/PD-L1 signaling and exerting antitumor effects *via*: (1) BIEXO reverses tumor cell malignancy; (2) IPA delivery enhances Tpex and effector T cells (Teff); (3) activated Teff-mediated cytotoxicity

Building upon these advances, we propose engineering iPSC-derived exosomes expressing anti-PD-1/MSLN single-chain variable fragments (scFv) loaded with IPA (BIEXO@IPA) to synergistically enhance CAR-T therapy in solid tumors (Scheme 1). This multifunctional platform simultaneously: (1) recruits PD-1^+^ T/CAR-T cells to MSLN-expressing tumors through bispecific engagement; (2) leverages inherent iPSC-exosome anti-tumor properties; (3) provides checkpoint blockade while preserving partial PD-1 function essential for T cell fitness; and (4) enhances Tpex proportion and function *via* localized IPA release, addressing the critical gap in metabolic support. This integrated approach represents the first reported IPA application for CAR-T enhancement, combining validated mechanisms with novel applications to comprehensively overcome the interconnected barriers preventing CAR-T success in solid tumors.

## Results

### Quality control of iPSC-derived exosomes

 To ensure translational rigor and minimize batch-to-batch variability, we established a standardized quality control workflow for iPSC-derived exosomes. Three independent batches demonstrated excellent reproducibility in physicochemical properties (CV < 10%; Table S1) and high purity (> 98%), as confirmed by SEC-HPLC (Figure S1a). Moreover, to verify the compositional consistency of the major exosomal cargo, we analyzed the top 500 most abundant proteins from the intersection of these three batches using LC-MS/MS. This analysis revealed a conserved core proteome that contained 70% of the top 100 canonical markers from the ExoCarta database (Figure S1b) [36]. To further elucidate the subcellular localization of the conserved proteome, Gene Ontology (GO) enrichment analysis was performed on the top 500 common proteins identified across three independent batches. As shown in Figure S1c, the proteins were significantly enriched in cellular components characteristic of exosomes, including extracellular exosome, extracellular vesicle, and plasma membrane raft. Specifically, this enrichment aligns with the known biogenesis pathway of exosomes [37]. This proteomic profiling confirms that the isolated iPSC-derived exosomes possess a stable and definitive exosomal identity, underscoring the robustness of our isolation process.

### Engineering and characterization of BIEXO@IPA

**Fig. 1:**
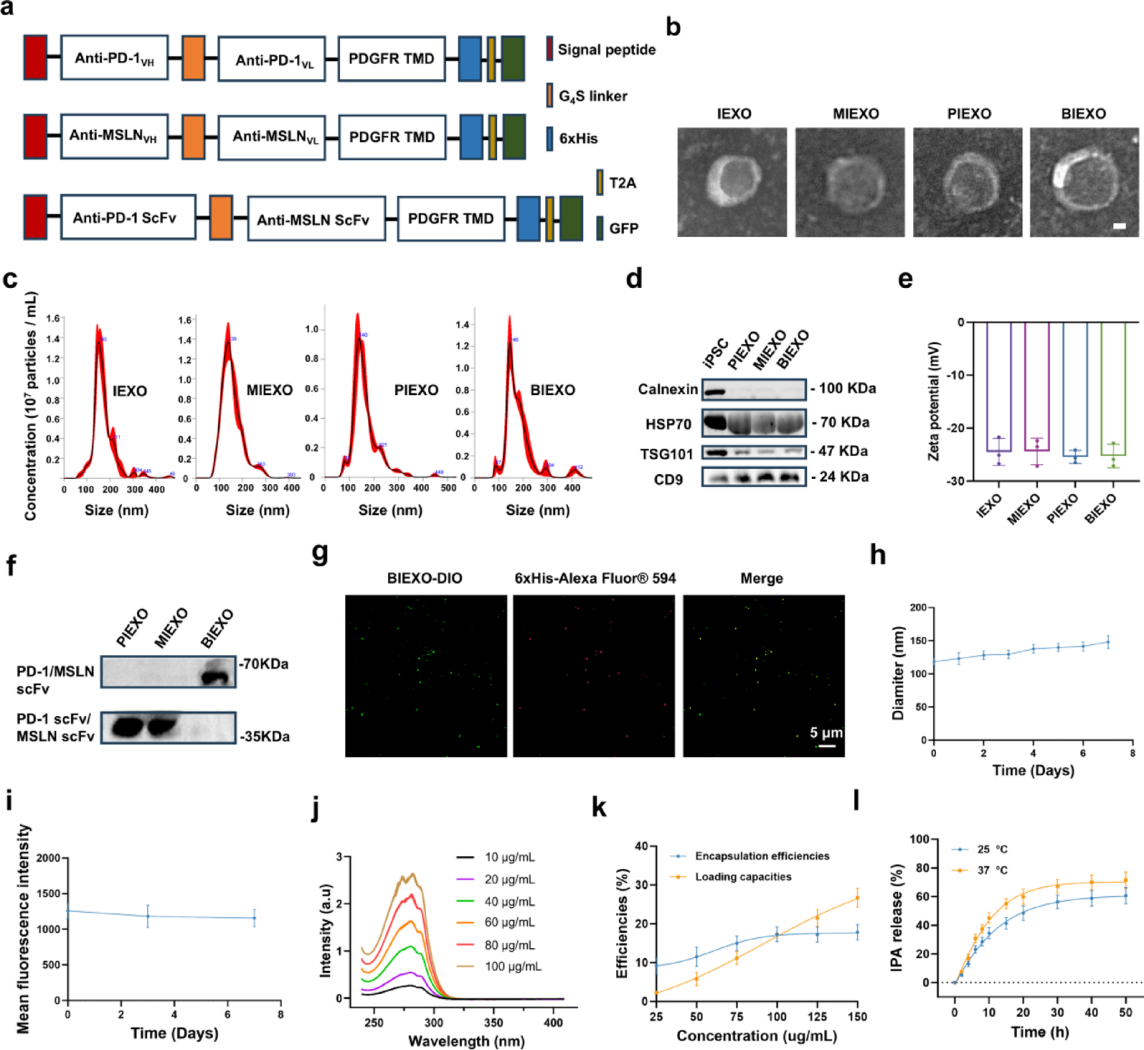
Engineering and characterization of BIEXO@IPA. (**a**) Schematic illustration of scFv constructs expressed on iPSCs. (**b-d**) Characterization of engineered exosomes by TEM (scale bar: 50 nm) (**b**), NTA (**c**) and western blotting of exosome markers (**d**). (**e**) Zeta potential measurements of engineered exosomes (*n* = 3). (**f**) Western blot of His-tagged scFv. (**g**) Immunofluorescence staining of His-tagged scFv on the BIEXO surface. (**h**) Size stability evaluation of BIEXOs incubated in 10% v/v serum-containing buffer over time (*n* = 3). (**i**) Stability assessment of bispecific antibodies (BsAbs) on the BIEXO surface *via* flow cytometry analysis after incubation in 10% v/v serum-containing buffer for 0, 3, and 7 days (*n* = 3). (**j**) Standard curve for IPA quantification by spectrophotometry, where IPA concentration (µg/mL) = 5.119 × OD value −8.469. (**k**) IPA encapsulation efficiency and loading capacity at various concentrations. (**l**) In vitro release profile of IPA from BIEXO in PBS (pH 7.4) at different temperatures, calculated as percentage release = (OD value of released IPA/OD value of total IPA in BIEXO) × 100%. The data are presented as mean ± SD

To develop a scalable platform for CAR-T enhancement, we first engineered iPSCs *via* lentiviral transduction to stably express anti-PD-1/MSLN bispecific scFv (BiPSC), along with monospecific constructs targeting either PD-1 (PiPSC) or MSLN (MiPSC) to serve as controls (Figure S2a, b). The scFv constructs were designed with the platelet-derived growth factor receptor (PDGFR) transmembrane domain to enable surface display on exosomes (Fig. 1a) [38, 39]. This strategy exploits the natural trafficking pathway of PDGFR through multivesicular bodies, directing the fusion proteins to the outer membrane of exosomes during biogenesis [40]. The transmembrane anchor ensures stable outward orientation of the scFv domains, maximizing their accessibility for receptor binding [41]. Previous studies have validated this PDGFR-based display system for various proteins including anti-EGFR scFv, anti-HER2/CD3 bispecific scFv, and immune checkpoint modulators, demonstrating its broad applicability for exosome surface engineering [42, 43]. Characterization revealed uniform nanovesicles (30–150 nm) with characteristic cup-shaped morphology (Fig. 1b, c). Zeta potential measurements showed similar negative charges (−20-30 mV; Fig. 1e). Western blotting and immunofluorescence confirmed successful scFv display (Fig. 1f, g). To further quantify the display efficiency, an indirect ELISA was performed. The results showed that the number of scFv molecules per exosome was 758 ± 57 for PIEXO, 785 ± 55 for MIEXO, and 732 ± 45 for BIEXO (Figure S3). The engineered exosomes demonstrated excellent stability, retaining > 90% of surface scFv expression after 7 days (Fig. 1h, i).

Notably, the parent iPSCs demonstrated exceptional exosome production capacity, yielding approximately 387.7-fold more exosomes than Lewis lung carcinoma (LLC) cells, 537.2-fold more than T cell-derived exosomes, and 14.8-fold more than mesenchymal stem cell (MSC)-derived exosomes per 10^7^ cells over 48 h (Figure S4a). These iPSC-derived exosomes (IEXOs) also exhibited intrinsic antitumor properties, inducing dose-dependent growth inhibition and apoptosis in LLC cells (Figure S4b-e), suppressing migration (Figure S4f, g), and modulating metastatic potential by decreasing vimentin expression while increasing apoptotic cleaved caspase-3 expression (Figure S4h). Transcriptomic analysis revealed significant downregulation of the expression of proliferation- and metastasis-associated genes following IEXO treatment (Figure S4i). These in vitro findings translated to significant in vivo efficacy, as intratumoral IEXO administration reduced tumor volume and weight compared with PBS controls in a subcutaneous LLC model (Figure S4j–l). These results highlight the multifaceted antitumor capabilities of IEXOs, potentially through reversal of malignant phenotypes.

For drug loading, we optimized IPA encapsulation at various concentrations. The 100 µg/mL concentration provided optimal balance between encapsulation efficiency and loading capacity (Fig. 1j, k). Release kinetics demonstrated sustained IPA delivery, with approximately 70% cumulative release at physiological temperature over 50 h (37 °C) (Fig. 1l). These findings collectively establish the successful engineering of stable, multifunctional exosomes with effective drug loading capabilities and suitable physicochemical properties for targeted cancer immunotherapy applications.

### Cellular Bridging and Potentiation of Antitumor Activity

**Fig. 2:**
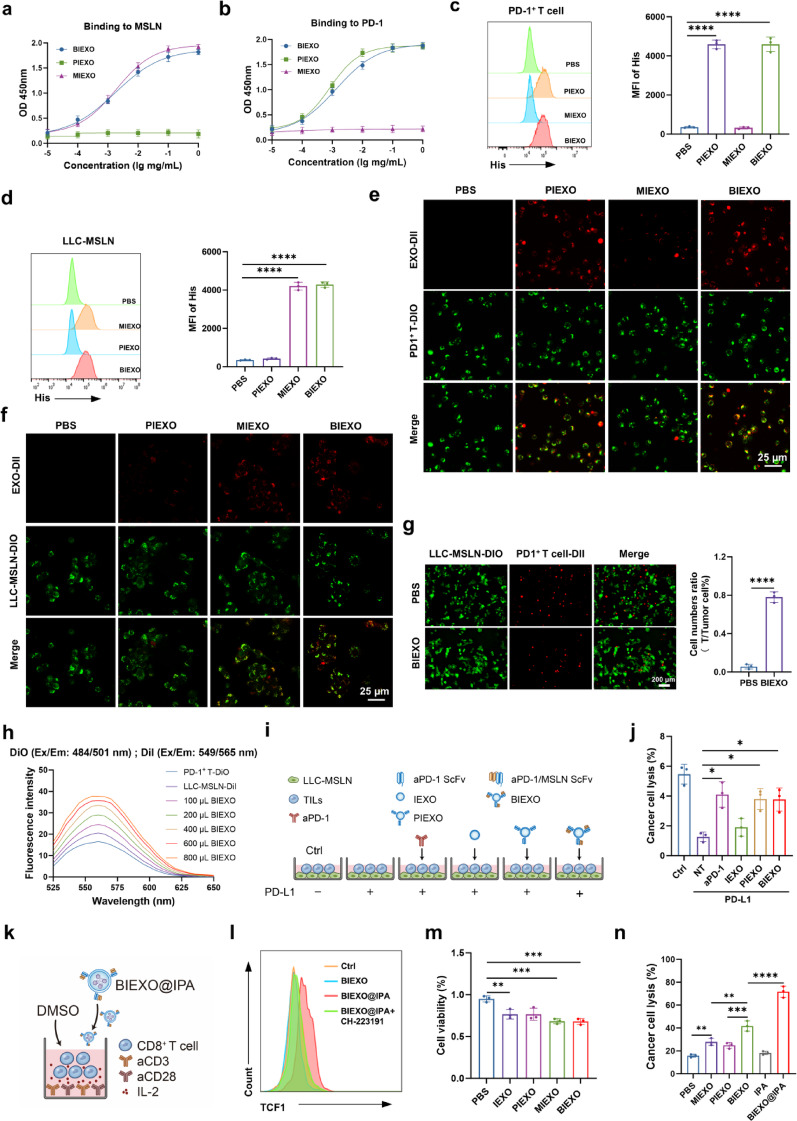
Functional characterization and T-cell bridging activity. (**a**,** b**) Binding affinity analysis of engineered exosomes (MIEXO, PIEXO, BIEXO) to MSLN (a) and PD-1 (**b**) *via* indirect ELISA. Exosomes were added to PD-1 or MSLN-coated plates and detected *via* an HRP-conjugated anti-His-tag antibody. (**c**,**d**) Flow cytometric analysis of engineered exosomes (100 µg/mL) binding to PD-1^+^ T cells (**c**) and LLC-MSLN cells (**d**), with His-tag mean fluorescence intensity (MFI) quantification (right panel). (**e**,** f**) Confocal microscopy analysis of BIEXO targeting to PD-1^+^ T cells (**e**) and LLC-MSLN cells (**f**). (**g**) Representative confocal microscopy images and corresponding quantification showing BIEXO-mediated bridging between PD-1^+^ T cells and LLC-MSLN cells. (**h**) FRET analysis of dose-dependent bridging efficiency. (**i**,** j**) Prevention of PD-L1-induced T-cell exhaustion measured by LDH release (*n* = 3). (**k**,** l**) Experimental design **(k)** for ex vivo culture of CD8^+^ T cells isolated from mouse spleen, and flow cytometric analysis of TCF-1^+^ Tpex cell expansion in PD-1^+^ CD8^+^ T cells following pre-treatment with the AhR antagonist CH-223,191 and BIEXO@IPA stimulation (**l**). (**m**) In vitro growth inhibition of LLC-MSLN cells following treatment. (**n**) Enhanced cytotoxicity of TILs isolated from LLC-MSLN tumor-bearing mice against LLC-MSLN cells following BIEXO@IPA treatment. Data are presented as mean ± SD. Statistical significance was determined using one-way ANOVA followed by Tukey’s multiple comparisons test (c, d, j, m, n). *p < 0.05, **p < 0.01, ***p < 0.001, ****p < 0.0001

Enzyme-linked immunosorbent assay (ELISA) confirmed concentration-dependent binding of BIEXO to both PD-1 and MSLN receptors (Fig. 2a, b). Flow cytometry validated specific receptor-mediated binding, with > 10-fold increased signal in target cells versus controls (Fig. 2c, d, S5). Confocal microscopy confirmed preferential binding to PD-1^+^ T cells and LLC-MSLN cells (Fig. 2e, f).

BIEXO effectively bridged PD-1^+^ T cells with LLC-MSLN cells, as demonstrated by microscopy and Förster resonance energy transfer (FRET) analysis, with dose-dependent enhancement of cell-cell proximity (Fig. 2g, h). Functionally, BIEXO prevented PD-L1-mediated T-cell exhaustion, maintaining cytotoxicity comparable to anti-PD-1 antibody (aPD-1) controls (Fig. 2i, j). Intratumoral administration of BIEXO@IPA significantly inhibited tumor growth compared with PBS group (Figure S6a). Remarkably, co-administration of exogenous PD-L1 did not compromise BIEXO@IPA efficacy, whereas PD-1 blocked BIEXO@IPA (Pb-BIEXO@IPA) treatment showed significantly attenuated tumor suppression in the presence of PD-L1, confirming that surface PD-1 confers resistance to PD-L1-mediated immunosuppression. Analysis of tumor-infiltrating lymphocytes revealed that BIEXO@IPA treatment maintained low PD-1 expression on CD8^+^ T cells even with exogenous PD-L1, whereas Pb-BIEXO@IPA + PD-L1 treatment resulted in elevated PD-1^+^ CD8^+^ T cells, indicative of enhanced exhaustion (Figure S6b). These findings establish BIEXO as a multifunctional immunotherapeutic platform that combines BiTE-like T cell engagement with intrinsic checkpoint blockade and cytokine neutralization capabilities. Importantly, compared with BIEXO alone, BIEXO@IPA treatment significantly expanded TCF-1^+^ cells within the PD-1^+^CD8^+^ population. Notably, this expansion was significantly abrogated by the AhR antagonist CH-223,191 (Fig. 2k, l). To further validate the activation of the AhR pathway, we examined the expression of its downstream targets, CYP1A1 and CYP1B1. Western blot analysis revealed that while BIEXO@IPA upregulated these proteins, CH-223,191 pre-treatment effectively inhibited their expression (Figure S7). Collectively, these results indicate that BIEXO@IPA preserves T-cell stemness in an AhR-dependent manner.

These molecular and cellular effects translated to functional antitumor outcomes. In vitro viability assays revealed that while IEXO inhibited LLC-MSLN proliferation compared with that of the PBS controls, BIEXO and MIEXO exhibited superior inhibitory effects, likely because enhanced tumor cell contact facilitated by their targeting capabilities (Fig. 2m). In the simulated tumor microenvironment, which incorporated tumor-infiltrating lymphocytes (TILs) isolated from LLC-MSLN tumor-bearing mice and LLC-MSLN cells, BIEXO induced significantly more potent tumor cytotoxicity than either PIEXO or MIEXO alone (Fig. 2n). When IPA was combined, the tumor killing efficacy was further enhanced, demonstrating the synergistic advantages of this combinatorial nanotherapeutic strategy in augmenting antitumor immune responses.

### Enhanced Pulmonary Delivery of BIEXO@IPA *via* Nebulized Administration

Conventional intravenous administration routes typically result in predominant hepatic accumulation of nanovesicles [44, 45], significantly limiting their therapeutic potential for extrahepatic malignancies. To overcome this limitation, we investigated nebulized pulmonary delivery as an alternative route for administering BIEXO@IPA in the context of lung cancer.

**Fig. 3:**
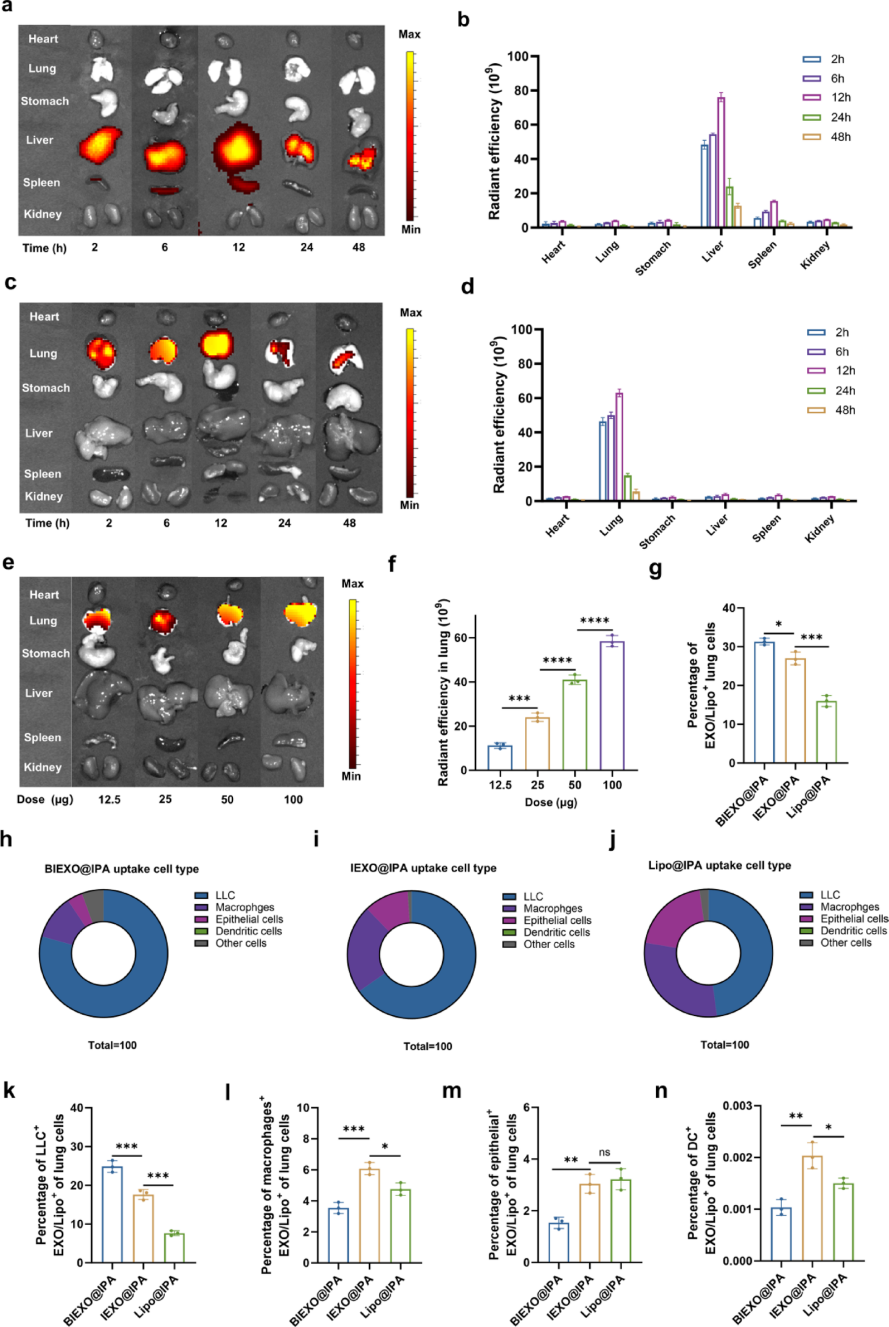
Biodistribution and cellular uptake of nebulized BIEXO@IPA. (**a-d**) In vivo biodistribution of DiR-labeled BIEXO@IPA following intravenous (i.v.) administration (a, b) or inhalation (inh.) (c, d), visualized by an IVIS imaging system at 2, 6, 12, 24 and 48 h post-administration (*n* = 3). (**e**,** f**) Dose-dependent pulmonary accumulation 12 h post-inhalation (*n* = 3). (**g**) Total lung cell uptake of BIEXO@IPA, IEXO@IPA, or Lipo@IPA by flow cytometry at 24 h post-inhalation (*n* = 3). (**h-j**) Comparative analysis of BIEXO@IPA, IEXO@IPA, and Lipo@IPA uptake distributions across different cell populations, presented as percentages of total uptake-positive cells (*n* = 3). (**k-n**) Flow cytometric analysis showing the uptake of BIEXO@IPA, IEXO@IPA, or Lipo@IPA by specific cell populations, including LLC-MSLN tumor cells (**k**), macrophages (F4/80^+^CD11b^+^) (**l**), epithelial cells (CD45^−^CD31^−^EpCAM^+^) (**m**), and dendritic cells (DCs, CD45^+^CD11c^+^CD24^+^) (**n**), expressed as a percentage of total lung cells at 24 h post administration (*n* = 3). The data are presented as the mean ± SD. Statistical significance was determined via one-way ANOVA followed by Tukey’s multiple comparisons test (**g**,** k-n**). *p < 0.05, **p < 0.01, ***p < 0.001. ns,* Not significant*

Intravenous injection resulted in predominant hepatic accumulation with minimal lung deposition, while nebulization achieved > 10-fold higher pulmonary accumulation (Fig. 3a-d). Dose-escalation studies demonstrated linear pulmonary accumulation across the therapeutic range (Fig. 3e, f).

We established an orthotopic lung cancer model using LLC-MSLN-luciferase cells to evaluate tumor-targeting specificity within the pulmonary environment (Figure S8). Initial IVIS studies demonstrated efficient colocalization of DIR-labeled BIEXO@IPA with lung tumor tissues, confirming its effective tumor-targeting potential (Figure S9a). Flow cytometry revealed hierarchical cellular uptake: BIEXO@IPA > IEXO@IPA > Lipo@IPA (31.33%, 26.75%, and 16.08% of total lung cells, respectively) (Fig. 3g). Critically, BIEXO@IPA achieved the highest tumor cell specificity (79.33% of internalized particles in LLC cells), compared to 65.26% for IEXO@IPA and 47.89% for Lipo@IPA (Fig. 3h-k), indicating that exosome-based delivery vehicles inherently possess superior uptake efficiency in lung cancer cells relative to liposomal formulations. Notably, the addition of bispecific antibodies to the exosome surface (BIEXO vs. IEXO) further enhanced the tumor-specific targeting efficiency by approximately 14%, clearly demonstrating the advantages and biological efficacy of the bispecific antibody strategy (Fig. 3g–j). By comparison, Lipo@IPA demonstrated poor tumor cell-targeting specificity, with less than 50% uptake occurring in LLC cells and most uptake observed in macrophages and epithelial cells (Fig. 3j–n). Minimal internalization (< 1%) in dendritic cells was observed across all formulations (Fig. 3n). In addition, pharmacokinetic analysis revealed that BIEXO exhibited biphasic clearance kinetics with an elimination half-life of 340 min, representing a 2.6-fold increase compared to the 133-min half-life of soluble anti-PD-1/MSLN BsAb (Figure S9b). This extended circulation time enhances tumor accumulation and sustains therapeutic activity.

Furthermore, transmembrane permeability assays validated the mucus-penetrating potential of BIEXO@IPA, addressing a critical barrier in pulmonary drug delivery (Figure S10). Notably, the intrinsic anionic surface characteristics minimized electrostatic entrapment within the negatively charged mucus environment.

### BIEXO@IPA Demonstrates Superior Antitumor Efficacy in Orthotopic Lung Cancer Model and Reprograms the Tumor Immune Microenvironment

**Fig. 4:**
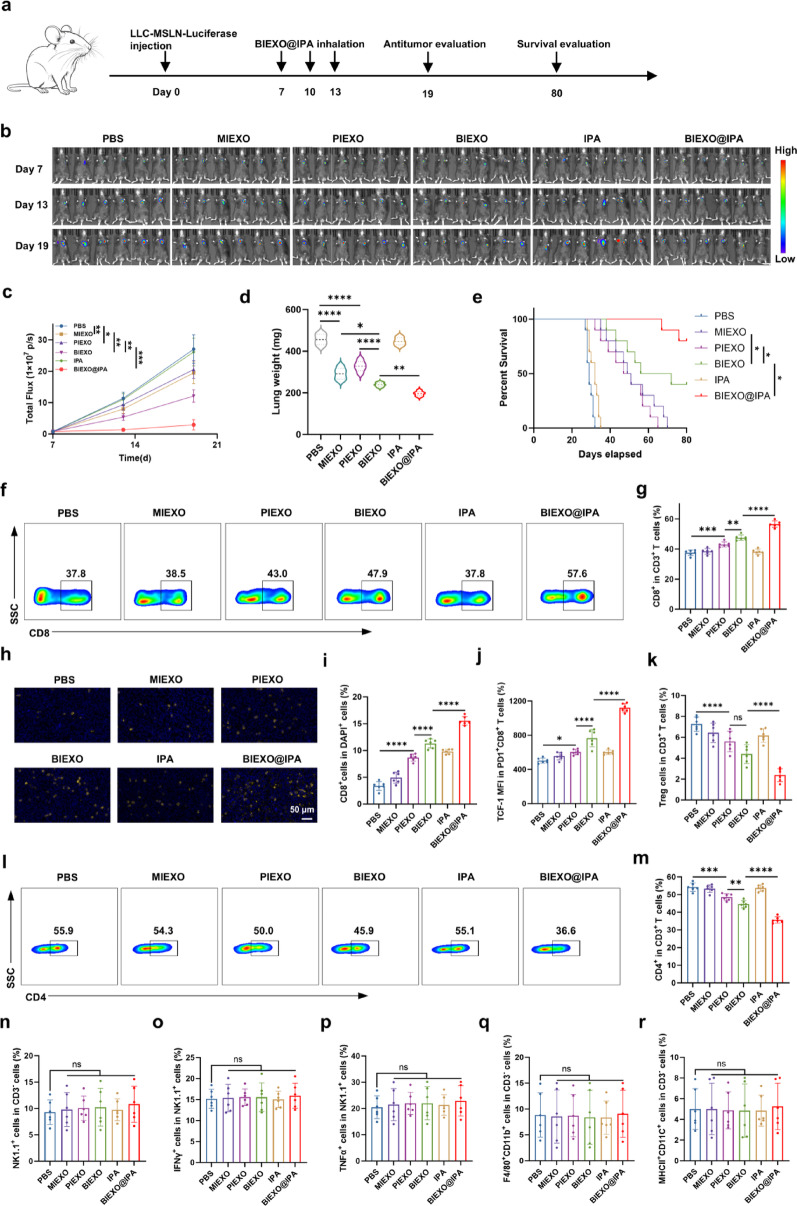
Therapeutic efficacy of nebulized inhalation of BIEXO@IPA in orthotopic lung cancer models and analysis of the tumor microenvironment. (**a**) Schematic illustration of the experimental design for BIEXO@IPA treatment. (**b**,** c**) Representative IVIS imaging of tumor progression over time in an orthotopic lung cancer model treated with inhaled BIEXO@IPA (**b**) and quantification of bioluminescence signals (**c**) (*n* = 6). (**d**) Lung volumes in each treatment group. (**e**) Kaplan-Meier survival curves. (**f**,** g**) Flow cytometric analysis of CD8⁺ T cell frequencies in orthotopic LLC tumors, showing representative plots (**f**) and quantification (**g**) (*n* = 6). (**h**,** i**) Immunofluorescence analysis of tumor-infiltrating CD8⁺ T cells, with representative images (**h**) and quantification (**i**) (*n* = 6). (**j**) TCF-1 expression levels in PD-1⁺CD8⁺ T cells (*n* = 6). (**k**) Frequency of Tregs among intratumoral CD3⁺ T cells (*n* = 6). (**l**,** m**) Flow cytometric analysis of CD4⁺ T cell frequencies in orthotopic LLC tumors, showing representative plots (**l**) and quantification (**m**) (*n* = 6). (n-r) The frequencies of distinct immune cell populations were quantified: NK1.1^+^ natural killer cells (**n**); IFN-γ-producing NK1.1^+^ cells (**o**); TNF-α-producing NK1.1^+^ cells (**p**); F4/80^+^CD11b^+^ macrophages (**q**); and MHCII^+^CD11c^+^ dendritic cells (**r**) (*n* = 6). Data are presented as mean ± SD. Statistical significance was determined by one-way ANOVA with Tukey’s multiple comparisons test (**d**,** g**,** i**,** j-r**), two-way ANOVA with Tukey’s multiple comparisons test (c) (significance annotations are shown for the final time point), and Log-rank (Mantel-Cox) test for survival analysis (e). *p < 0.05, **p < 0.01, ***p < 0.001, ****p < 0.0001; ns,* not significant*

Building on the distribution findings, we systematically evaluated the therapeutic efficacy of BIEXO@IPA in orthotopic lung cancer model established by LLC-MSLN-luciferase cells and administered nebulized treatments (Fig. 4a). Longitudinal bioluminescence imaging revealed remarkable tumor burden reduction with BIEXO@IPA, achieving an 87.9% decrease compared to PBS controls, while BIEXO monotherapy demonstrated a 49.1% reduction. Notably, all of the BIEXO@IPA-treated animals maintained a minimal tumor burden throughout the study period, while both the MIEXO and PIEXO groups showed modest therapeutic benefits compared with the PBS control group, suppressing tumor burden by 17.1% and 9.7%, respectively (Fig. 4b-d). This progressive enhancement in therapeutic efficacy translated directly to survival outcomes. Kaplan‒Meier analysis revealed that BIEXO@IPA treatment resulted in 80% of the mice surviving beyond the 80-day study endpoint. In contrast, BIEXO monotherapy achieved 40% long-term survival, whereas PIEXO and MIEXO yielded median survival times of 49 and 50 days, respectively, with no long-term survival. The control groups presented markedly reduced survival; IPA alone resulted in a median survival of 32 days, and the PBS controls succumbed at a median of 29 days (Fig. 4e).

To elucidate the immunological mechanisms underlying the superior efficacy of BIEXO@IPA, we performed comprehensive flow cytometric analyses of tumor-infiltrating immune populations in the orthotopic model on day 14 after treatment initiation. BIEXO@IPA treatment dramatically increased the proportion of CD8^+^ T cells within the TME (Fig. 4f, g). Immunofluorescence staining of tumor sections further corroborated these findings, confirming enhanced CD8^+^ T-cell infiltration following treatment (Fig. 4h, i). More importantly, these tumor-infiltrating CD8^+^ T cells presented significantly enhanced effector functions, with substantial increases in IFN-γ^+^, TNF-α^+^, and Granzyme B^+^ populations (Figure S11a-f). We observed that, compared with BIEXO alone, BIEXO@IPA treatment significantly increased the proportion of TCF-1^+^ cells among PD-1^+^CD8^+^ T cells, suggesting a potential synergistic effect of the IPA component (Fig. 4j). BIEXO@IPA treatment also significantly reduced CD4^+^ T cell infiltration, particularly the regulatory T cell compartment (Fig. 4k-m). Notably, PIEXO induced modest immunological changes, while BIEXO produced intermediate effects between those of single-targeted formulations and BIEXO@IPA. Despite these marked T-cell alterations, we observed no significant changes in natural killer cells, dendritic cells, or macrophage populations (Fig. 4n-r).

Immune cell depletion studies definitively established that the therapeutic efficacy was T cell-dependent, with CD8^+^ T cells playing the predominant antitumor role. CD4^+^ T cells contributed partially to the therapeutic effect, while NK cells and macrophages demonstrated negligible impact on treatment outcomes (Figure S12). These findings collectively establish that the superior antitumor efficacy of BIEXO@IPA stems primarily from targeted enhancement of cytotoxic T cell recruitment and functional activation within the tumor microenvironment.

### Biosafety Profile of BIEXO@IPA

**Fig. 5:**
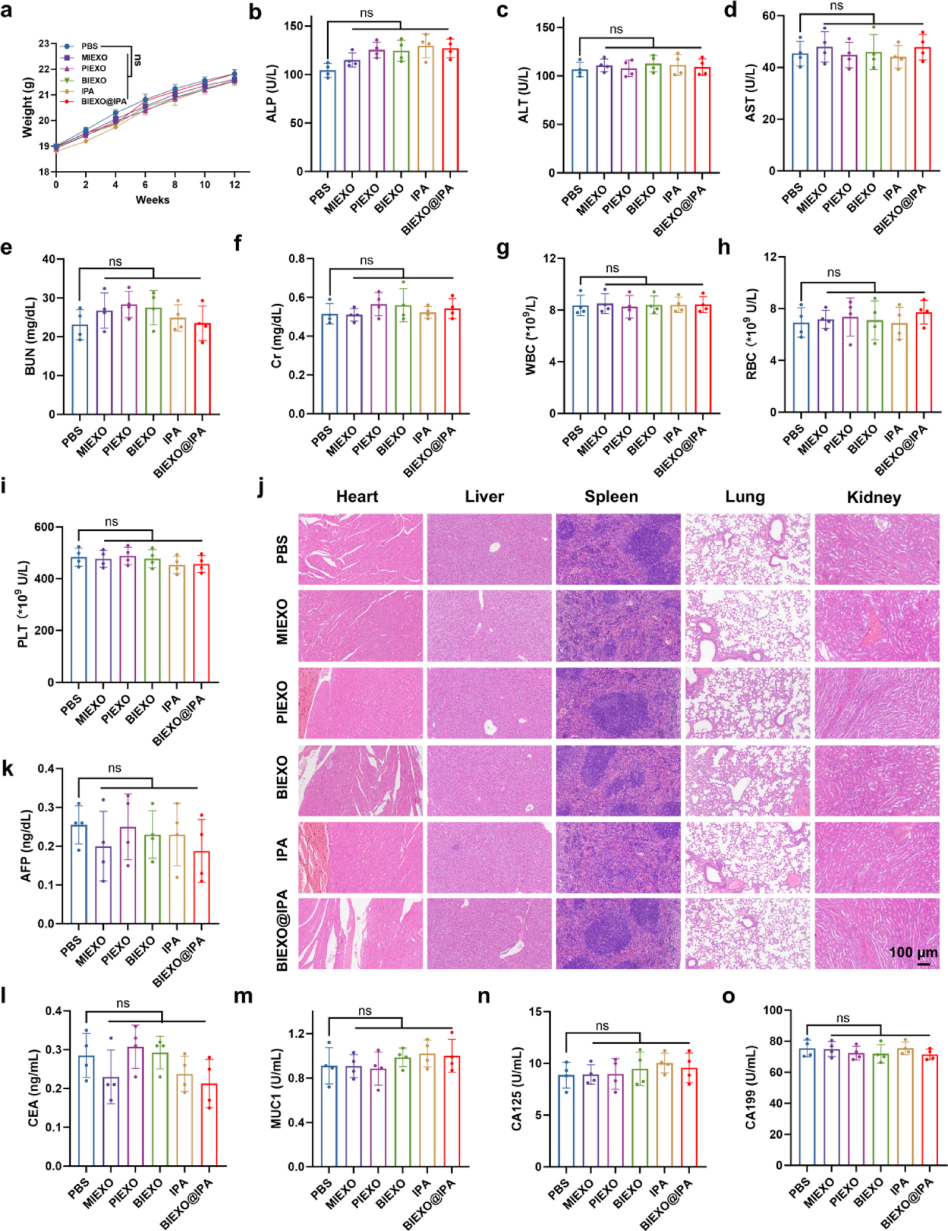
Biocompatibility of BIEXO@IPA. (**a**) Body weight monitoring of mice following BIEXO@IPA inhalation for 12 weeks. (**b–i**) Serum biochemical and hematological parameters measured 30 days after BIEXO@IPA treatment: ALP (**b**), ALT (**c**), AST (**d**), BUN (**e**), Cr (**f**), WBC (**g**), RBC (**h**), and PLT (**i**) (*n* = 4). (**j**) Representative hematoxylin and eosin (H&E) staining of tissue sections from the heart, liver, spleen, lung, and kidney to assess potential systemic toxicity. (**k–o**) Serum levels of tumor markers in mice across different treatment groups: AFP (**k**), CEA (**l**), MUC1 (**m**), CA125 (**n**), and CA19-9 (**o**) (*n* = 4). Data are presented as mean ± SD. Statistical significance was determined by two-way ANOVA with Tukey's multiple comparisons test (**a**), or one-way ANOVA followed by Tukey’s multiple comparisons test (**b–i**, **k–o**); ns, not significant

To evaluate BIEXO@IPA biocompatibility, we monitored body weights in mice over 12 weeks, revealing no intergroup differences (Fig. 5a). In vitro assays showed negligible cytotoxicity toward normal hepatocytes, lung epithelial cells, and renal cells (Figure S13). Serum biochemical markers, including liver enzymes alkaline phosphatase (ALP), alanine aminotransferase (ALT), and aspartate aminotransferase (AST), as well as kidney function indicators blood urea nitrogen (BUN) and creatinine (Cr), remained within normal ranges (Fig. 5b-f). Hematological parameters including platelet (PLT), red blood cell (RBC), and white blood cell (WBC) showed no treatment-related alterations (Fig. 5g-i). Histopathological examination revealed no organ toxicity (Fig. 5j). The results revealed no significant differences between the BIEXO@IPA and PBS control groups.

Given the iPSC origin of our exosomes, we specifically addressed potential tumorigenicity concerns—a critical safety consideration for pluripotent stem cell-derived products [46, 47]. Serum tumor marker panel analysis, encompassing alpha-fetoprotein (AFP), carcinoembryonic antigen (CEA), mucin 1 (MUC1), cancer antigen 125 (CA125), and carbohydrate antigen 19 − 9 (CA19-9), demonstrated no elevation compared to baseline levels (Fig. 5k-o). Collectively, these comprehensive safety evaluations establish BIEXO@IPA as a biocompatible therapeutic platform with favorable in vivo tolerability.

### BIEXO@IPA Augments CAR-T Antitumor Efficacy in Lung Cancer Models

**Fig. 6:**
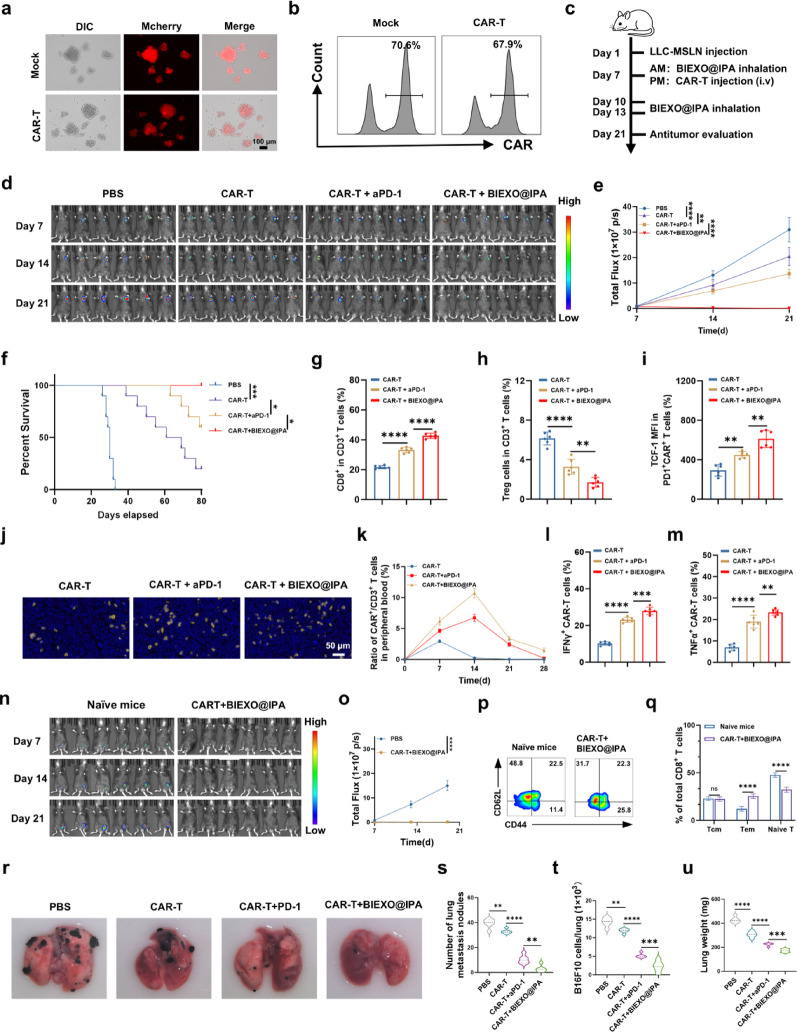
BIEXO@IPA augments CAR-T antitumor efficacy in lung cancer models. (**a**) Representative fluorescence microscopy images of mCherry-labeled CAR-T cells following lentiviral transduction. (**b**) Flow cytometric quantification of CAR expression frequency in primary T cells. (**c**) Schematic illustration of the treatment schedule for combined BIEXO@IPA and CAR-T cell therapy. (**d**,** e**) Longitudinal monitoring of orthotopic tumor progression *via* bioluminescence imaging (**d**) and quantification of total flux (**e**) across treatment groups (*n* = 6). (**f**) Kaplan-Meier survival analysis of tumor-bearing mice in different treatment cohorts. (**g-i**) Comprehensive intratumoral immune profiling after therapy: frequency of tumor-infiltrating CD8⁺ T cells among CD3⁺ cells (**g**), proportion of CD4⁺FOXP3⁺ regulatory T cells (Tregs) among CD3⁺ T cells (**h**), and median fluorescence intensity of TCF-1 in tumor-infiltrating CAR-T cells (**i**) (*n* = 6). (**j**) Immunofluorescence analysis of CAR-T cell infiltration in tumor tissue sections (blue: DAPI; yellow: myc). (**k**) Persistence kinetics of CAR-T cells in peripheral blood at indicated time points post-adoptive transfer in LLC-MSLN tumor-bearing mice (*n* = 6). (**l**,** m**) Frequency of IFN-γ⁺ (**l**) and TNF-α⁺ (**m**) cells among tumor-infiltrating CAR-T cells (*n* = 6). (**n**,** o**) Long-term tumor immunity assessment in mice achieving complete tumor remission following BIEXO@IPA/CAR-T combination treatment. Mice were rechallenged with LLC-MSLN-luciferase cells on day 110 post-tumor inoculation, with subsequent quantification of tumor bioluminescence on day 21 post-rechallenge (**n**) and corresponding quantitative analysis (**o**) (*n* = 6). (**p**,** q**) Characterization of immune memory response in mice exhibiting complete tumor regression. PBMCs were isolated following in vivo restimulation with LLC-MSLN-luciferase cell lysates, and the proportion of memory T cells was quantified *via* flow cytometry (*n* = 6). (**r-u**) Evaluation of combined BIEXO@IPA/CAR-T efficacy in a B16F10-MSLN experimental lung metastasis model: representative macroscopic images of lung metastatic burden (**r**), quantification of visible metastatic nodules (**s**), flow cytometric enumeration of B16F10 cells in lung tissue (**t**), and lung weight measurements (**u**) across different treatment groups (*n* = 6). Data are presented as mean ± SD. Statistical significance was determined by log-rank test (**b**), two-way ANOVA with Tukey’s post-hoc test (**e**,** o**) (significance annotations for these time-course experiments are shown for the final time point), or one-way ANOVA with Tukey’s post-hoc test (**g-i**,** l**,** m**,** q**,** s-u**). *p < 0.05, **p < 0.01, ***p < 0.001, ****p < 0.0001; ns,* not significant*

Building on our demonstration that BIEXO@IPA potentiates T cell-immunity, we next investigated its potential to augment the antitumor activity of CAR-T cells in an orthotopic lung cancer model. For this purpose, we employed a lentiviral vector engineered to co-express a Myc-tagged anti-MSLN CAR along with a distinct mCherry reporter for cellular tracking, which was subsequently used to transduce murine T cells (Fig. 6a–b). Mice bearing established orthotopic lung tumors received inhaled BIEXO@IPA concomitant with CAR-T cell injection; PBS, CAR-T alone and CAR-T plus aPD-1 served as comparators (Fig. 6c). Although CAR-T plus aPD-1 provided modest benefit, the combination of BIEXO@IPA inhalation and CAR-T therapy achieved superior tumor control. In the tumor progression study, 66.7% of animals attained complete remission, as monitored by longitudinal IVIS imaging (Fig. 6d, e). This translated to 100% survival at day 80 in the CAR-T + BIEXO@IPA group, compared to only 33.3% with CAR-T + aPD-1 and 16.7% with CAR-T monotherapy (Fig. 6f). All PBS-treated mice succumbed by day 33 with a median survival of 30 days.

Mechanistic analysis revealed profound immunological reprogramming by BIEXO@IPA. Intratumoral CD8^+^ T cell frequency doubled from 21.4% with CAR-T alone to 42.9% with CAR-T + BIEXO@IPA, while CAR-T + aPD-1 achieved only intermediate enhancement to 33.3% (Fig. 6g). Regulatory T cells decreased by 75% with BIEXO@IPA combination, compared to 54% reduction with aPD-1 (Fig. 6h). Critically, TCF-1 expression in PD-1^+^ CAR-T cells increased 2.3-fold with BIEXO@IPA, surpassing the 1.6-fold increase observed with aPD-1, indicating superior stem-like memory formation (Fig. 6i). Immunofluorescence confirmed deepest CAR-T penetration throughout tumor parenchyma following BIEXO@IPA treatment (Fig. 6j).

Longitudinal monitoring of peripheral blood showed that CAR-T + BIEXO@IPA achieved a peak CAR-T frequency of 10.7% of total T cells at day 14—representing 3.6-fold and 1.4-fold improvements over monotherapy and aPD-1 combination, respectively (Fig. 6k). Concordantly, the percentages of intratumoral CAR-T cells expressing IFN-γ and TNF-α were significantly elevated in the BIEXO@IPA + CAR-T group (Fig. 6l, m). Cytokine profiling of serum and bronchoalveolar lavage fluid (BALF) revealed that, compared to CAR-T alone, CAR-T + BIEXO@IPA treatment resulted in elevated levels of IL-6, TNF-α, and IFN-γ in the lung, with only mild increases in serum, suggesting a low risk of cytokine release syndrome (Figure S14). These data establish that, as an application extension of its immune-activating properties, inhaled BIEXO@IPA robustly enhances CAR-T cell infiltration, persistence, and effector function in lung cancer, translating into superior antitumor efficacy in vivo.

To assess immune memory, mice achieving complete remission were rechallenged with tumor cells at day 110. Remarkably, 83.3% resisted secondary tumor growth, while naive controls developed progressive tumors (Fig. 6n, o). Flow cytometric analysis revealed expansion of effector memory T cells and reduction of naive populations, confirming durable immune memory establishment (Fig. 6p, q).

To assess therapeutic versatility, we evaluated BIEXO@IPA in a B16F10-MSLN melanoma pulmonary metastasis model (Figure S15). Following intravenous tumor cell injection, mice received inhaled treatments combined with CAR-T therapy. BIEXO@IPA demonstrated superior antimetastatic efficacy across all treatment groups. While CAR-T monotherapy achieved modest reduction in metastatic burden (17% reduction vs. PBS), CAR-T + aPD-1 showed substantial improvement (73% reduction). Most remarkably, CAR-T + BIEXO@IPA nearly eliminated pulmonary metastases with 93% reduction, significantly surpassing the anti-PD-1 combination (Fig. 6r-s).

To further bridge the gap toward clinical translation, we validated the efficacy of BIEXO@IPA in a humanized model using patient-derived primary lung cancer cells. We engineered these primary cells to stably express MSLN-GFP (Figure S16a) and confirmed the generation of functional CAR-T cells (Figure S16b). Notably, in vitro LDH release assays revealed that CAR-T cells combined with BIEXO@IPA exhibited significantly stronger killing effects against primary lung cancer cells compared to CAR-T monotherapy or the combination with anti-PD-1 antibody (Figure S16c). Subsequently, we established orthotopic PDX models to evaluate in vivo therapeutic potential. IVIS imaging revealed that the combination of BIEXO@IPA and CAR-T cells exerted superior tumor growth suppression compared to controls (Figure S16d, e). This enhanced tumor control translated into a significant survival advantage (Figure S16f), underscoring the platform’s potential for clinical application.

These compelling results across orthotopic, rechallenge, metastatic, and humanized PDX models establish that BIEXO@IPA surpasses conventional PD-1 blockade in augmenting CAR-T therapy, representing a superior strategy for treating both primary and metastatic pulmonary malignancies.

## Discussion

In this study, we introduced a novel inhalable nanoplatform utilizing induced pluripotent stem cell (iPSC)-derived exosomes (IEXOs) as biocompatible nanocarriers, engineered for co-delivery of bispecific PD-1/mesothelin T cell engagers and IPA to reprogram T cell metabolism. This approach synergistically augmented CAR-T therapy in lung cancer models by overcoming T cell exhaustion, infiltration deficits, and immunosuppressive tumor microenvironments. Our findings highlight IEXOs’ intrinsic antitumor effects and scalable production, culminating in marked in vivo efficacy, including high rates of tumor regression and prolonged survival. These outcomes position the platform as a bioinspired nanoparticle system for targeted pulmonary delivery, leveraging exosomes’ nanoscale size, biocompatibility, and controlled release kinetics.

Mechanistically, our engineered exosomes (BIEXOs) function through a multimodal approach. The bispecific scFv bridges CAR-T cells to MSLN-expressing tumor cells while blocking PD-1 checkpoints, promoting T cell activation and infiltration into the TME. Concurrently, IPA-loaded exosomes likely reprogram T cell metabolism by activating the aryl hydrocarbon receptor (AhR) pathway as reported in previous studies [12, 37], which is consistent with our observation of expanded TCF-1^+^ Tpex populations. Notably, iPSC-derived exosomes contribute intrinsic anti-tumor activity beyond their role as delivery vehicles, directly suppressing tumor cell proliferation and invasion. This multifaceted functionality distinguishes our platform from conventional synthetic nanocarriers that merely serve as passive delivery vehicles.

From a bioengineering perspective, iPSC-derived exosomes exhibit significantly superior characteristics compared to traditional cell-derived exosomes, including greater scalability, amenability to genetic modification, and intrinsic antitumor properties. These advantages establish a next-generation nanotherapeutic platform with unprecedented versatility. The exosomes’ surface engineering *via* PDGFR display and electroporation loading ensures targeted delivery and sustained IPA release, with zeta potentials of −20 to −30 mV facilitating mucus penetration in aerosolized formulations. These features exploit exosomes’ natural biogenesis from multivesicular bodies, positioning them as scalable, biologically derived nanovesicles superior to synthetic liposomes [28, 29, 48, 49]. 

Compared to existing therapies, our platform addresses limitations of traditional bispecific T cell engagers (BiTEs), which suffer from short half-lives and potential Treg recruitment [19–23]. Prior studies on exosome-based immunotherapy have focused on tumor-derived exosomes or simple BiTEs [50, 51]. Our integration of iPSC-derived exosomes with BiTEs and IPA represents a first-in-kind approach for solid tumors. For instance, while IPA has shown promise in gut microbiome-linked immunomodulation [26, 27], its unexplored role in CAR-T enhancement aligns with recent immunology advances emphasizing Tpex metabolism [24, 25]. Our inhalation route outperforms systemic delivery in lung-specific models, achieving > 10-fold higher pulmonary accumulation than intravenous administration, consistent with nanoparticle aerosolization studies [52–55]. This cross-disciplinary fusion of nanomedicine, immunology, and drug delivery highlights the translational edge over lipid nanoparticle systems, which often lack intrinsic antitumor cargo.

In comparison with existing clinical strategies, our BIEXO@IPA platform demonstrates several distinct advantages over current clinical approaches: (1) Versus Anti-PD-1 Monoclonal Antibodies: While anti-PD-1 antibodies (e.g., pembrolizumab, nivolumab) have revolutionized cancer immunotherapy, their combination with CAR-T cells in solid tumors often shows variable efficacy due to the complex immunosuppressive tumor microenvironment [1, 8]. In our orthotopic model, the standard anti-PD-1 combination achieved only modest survival benefits (~33% survival), whereas BIEXO@IPA resulted in 100% survival. This superior efficacy is likely attributed to the platform’s dual mechanism: localized delivery ensures high intratumoral concentration, while IPA-mediated metabolic reprogramming provides a synergistic boost to T cell stemness that simple checkpoint blockade cannot offer [26]. (2) Versus BiTE Drugs: Clinically approved BiTEs (e.g., blinatumomab) face significant pharmacological hurdles, primarily their short half-life which necessitates continuous intravenous infusion [21]. Furthermore, systemic BiTE administration carries non-negligible risks of cytokine release syndrome (CRS) and potential Treg recruitment [51]. Our study demonstrates that displaying BiTEs on the exosome surface significantly extends the functional half-life to 340 min (vs. 133 min for soluble BsAb). Furthermore, our inhalable delivery achieves potent effector cytokine production within the tumor microenvironment while maintaining minimal systemic cytokine elevation, addressing the critical safety limitations of systemic BiTE therapy [52, 53]. (3) Immunological Profiling Comparison: The depth of immune remodeling further distinguishes our platform from standard checkpoint blockade. In the context of CAR-T combination, BIEXO@IPA induced a significantly greater expansion of stem-like TCF-1⁺ Tpex cells (2.3-fold increase) compared to the anti-PD-1 combination (1.6-fold). Additionally, it achieved a deeper depletion of immunosuppressive Tregs (75% vs. 54% reduction). This data indicates that BIEXO@IPA functions not merely as a checkpoint inhibitor, but as a comprehensive immunometabolic modulator that creates a more favorable microenvironment for CAR-T persistence.

Despite these advancements, our study has limitations. While iPSC exosome production is highly scalable, batch-to-batch variability could arise from reprogramming efficiency or culture conditions, necessitating good manufacturing practice (GMP) optimization. Furthermore, our models relied on syngeneic mice, limiting direct extrapolation to human heterogeneity. Future work should incorporate humanized models, such as patient-derived xenografts or organoids, to evaluate cross-species efficacy and advance toward clinical trials. Long-term studies on exosome biodistribution and combination with other immunotherapies could further refine this platform.

In summary, this iPSC-exosome nanoplatform offers a promising strategy for augmenting CAR-T therapy in lung cancer, with high translational potential due to its scalability, safety profile, and multimodal synergy. By bridging nanotechnology with immunology, it paves the way for next-generation inhalable therapeutics.

## Experimental design

### Mice

C57BL/6J mice (aged 6–8 weeks) were acquired from the Guangdong Experimental Animal Center. This study was approved by the Animal Ethics Committee of the Fifth Affiliated Hospital of Sun Yat-sen University (approval number: 00560). All of the animal experiments were conducted in accordance with the National Institutes of Health Guide for the Care and Use of Laboratory Animals and the Animal Care and Use Committee protocols of Sun Yat-sen University.

### Cell lines and culture

Human embryonic kidney 293 T cells, LLC, and B16F10 cells were obtained from ATCC and cultured in Dulbecco’s Modified Eagle’s Medium (DMEM, Thermo Fisher Scientific) supplemented with 10% fetal bovine serum (FBS, Invitrogen). Mouse spleen T cells were isolated by fluorescence-activated cell sorting (FACS) using anti-CD3-APC antibody (BioLegend), and cultured in serum-free medium (Basso Cell Technology) with 150 U/mL recombinant IL-2. Tumor cells isolated from mice bearing subcutaneous LLC tumors were expanded in DMEM +10% FBS. IPSCs from C57BL/6 mice were prepared as described in our previous study [56, 57], and maintained on gelatin-coated plates in Oricell mESC serum-free medium (Cyagen). All cultures tested negative for mycoplasma.

### Plasmid Construction and Lentiviral Transduction

Anti-MSLN scFv (derived from human P4 antibody) and anti-PD-1 scFv (from murine RMP1-14 antibody for syngeneic models or the human pembrolizumab antibody for humanized validation) were connected *via* a (GGGGS)₃ linker [58, 59]. The PDGFRβ transmembrane domain (amino acids 514–562 of human PDGFRβ) was inserted between the scFv sequence and the C-terminal His-tag, providing type I membrane protein topology with extracellular scFv display [38, 39]. These sequences were cloned into pCDH-EF1 lentiviral vector.

Lentiviral particles were produced by co-transfecting 293 T cells with the expression vector and packaging plasmids (pMD2.G and psPAX2) at a 4:1:3 molar ratio (expression vector: pMD2.G: psPAX2) using polyethylenimine (PEI). Viral supernatants were collected 48–72 h post-transfection, filtered through 0.45 μm filters, and used to transduce iPSCs at a multiplicity of infection (MOI) of 5. Stable cell lines expressing anti-MSLN scFv (MiPSC), anti-PD-1 scFv (PiPSC), or anti-PD-1/MSLN scFv (BiPSC) were selected using puromycin (2 µg/mL). Similarly, LLC and B16F10 cells were transduced to generate LLC-MSLN-luciferase and B16F10-MSLN cell lines, respectively. Patient-derived primary lung cancer cells were transduced with lentivirus encoding MSLN-Luciferase-GFP and isolated via FACS based on GFP expression.

### Preparation and characteristics of MSLN CAR-T cells

Anti-MSLN CAR construct was cloned into pLV-EF1α-Myc-mCherry lentiviral vector. Lentiviral particles were produced as described above using a 4:1:3 molar ratio of pLV-MSLN-CAR: pMD2.G: psPAX2. Murine splenic T cells were activated with anti-CD3 (1 µg/mL) and anti-CD28 (2 µg/mL) antibodies (PeproTech) for 24 h, whereas human T cells (isolated from lung cancer patient PBMCs) were activated with anti-CD3/CD28 Dynabeads (Thermo Fisher Scientific) for 48 h, then transduced at an MOI of 5. Seven days post-transduction, CAR expression was detected by flow cytometry using Protein L (1 µg/mL, 30 min, 4 °C) followed by FITC-streptavidin (BioLegend). Data were acquired on a CytoFLEX LX cytometer (Beckman Coulter).

### Preparation of Exosomes

Exosomes were isolated from culture supernatants of iPSC, MiPSC, PiPSC, and BiPSC using differential ultracentrifugation [60]. Briefly, supernatants were sequentially centrifuged at 800 × g (5 min), 2,000 × g (10 min), and 10,000 × g (30 min) to remove cells and debris. The clarified supernatant was passed through a 0.22 μm sterile membrane filter and ultracentrifuged at 100,000 × g for 90 min at 4 °C (Beckman Coulter Optima XPN-100). Exosome pellets were washed in PBS and re-ultracentrifuged under identical conditions.

### Nanoparticle tracking analysis

Nanoparticle tracking analysis was performed *via* a NanoSight NS400 (Malvern Instruments Company, Malvern, UK) to assess the concentration and size distribution of the isolated exosomes. A freshly concentrated exosome mixture was introduced into the sample chamber *via* a sterile injection syringe, ensuring constant flow during the injection process to maintain sample integrity. The data obtained were subsequently analyzed *via* NTA 3.0 software (Malvern).

### Transmission electron microscopy

Transmission electron microscopy was employed to investigate the morphology of the isolated exosomes following established protocols [61]. For this analysis, a 10 µL suspension of exosomes was prepared and subjected to negative staining with 2% uranyl acetate. After allowing the grid to dry and remove excess fluid, the samples were examined under a transmission electron microscope operating at 120 kV, facilitating detailed visualization of the exosomal structures.

### Western blot assay of tumor-associated antigens

Exosomes were pelleted and washed twice in PBS, then lysed on ice for 15 min using lysis buffer. After centrifugation at 12,000 × g for 15 min at 4 °C, the exosomes were collected and their protein content quantified by BCA assay. Exosomal lysates were normalized to 20 µg per lane and separated on 12% SDS–PAGE gels. Proteins were transferred onto PVDF membranes (Millipore), which were blocked in 5% non-fat milk/TBST for 1 h at room temperature. Membranes were incubated overnight at 4 °C with the following primary antibodies (all at 1:1,000): anti-Calnexin (ab22595), anti-HSP70 (ab194360), anti-TSG101 (ab133586), anti-CD9 (ab223052) and anti-6×His tag (ab245114). After washing, blots were treated for 1.5 h at room temperature with HRP-conjugated anti-rabbit IgG (1:5,000; ab6721). Signal detection was carried out using the ECL-Plus chemiluminescence kit (Thermo Fisher Scientific).

### Quality Control and Proteomic Analysis of Exosomes

Batch-to-batch consistency was evaluated by measuring particle size and concentration via NTA, Zeta potential via Dynamic Light Scattering (DLS), and total protein yield via the BCA assay, with the CV calculated for all parameters to assess reproducibility. Purity was further analyzed by SEC-HPLC using a qEV column (Izon Science), monitoring absorbance at 214/280 nm to confirm the separation of vesicles from free proteins. For proteomic profiling, exosomal proteins were digested and analyzed by LC-MS/MS. To identify the robust core proteome, the top 500 most abundant proteins were selected from each of three independent batches, and the intersection of these datasets was determined to define the consensus high-abundance proteome, which was validated against the ExoCarta database [36] for identity confirmation.

### Quantification of Surface scFv on Engineered Exosomes by ELISA

ELISA 96-well plates were coated with various concentrations of recombinant 6×His protein (Sino Biological, 13105-S07E) and engineered exosomes (PIEXO, MIEXO, and BIEXO) overnight at 4 °C. Non-bound antigens were washed away with PBST (0.05% Tween 20) three times. The wells were blocked with PBS containing 2% BSA for 2 h, followed by washing with PBST. The rabbit anti-His polyclonal antibody (2 µg/mL; Abcam, ab9108) was subsequently added for 2-h incubation, followed by washing and incubation with an HRP-conjugated goat anti-rabbit IgG secondary antibody (BioLegend, 406401) for 1 h. TMB Substrate (Thermo Fisher Scientific, N301) was then added after washing, and the absorbance signals were measured at 450 nm using a BioTek Synergy H1 Hybrid Multi-Mode Microplate reader (BioTek, VT, USA). Given the 1:1 molar ratio of the His-tag to the scFv fusion protein, serial dilutions of recombinant 6×His-tagged protein standard in PBS were used as standards to determine the molar concentrations of surface scFv on engineered exosomes. The numbers of surface scFv per exosome particle were calculated based on the measured molar concentrations of surface scFv and the particle concentrations of the same batch of engineered exosomes by NTA.

### Loading of IPA into BIEXO

IPA (Sigma-Aldrich) was loaded into BIEXO *via* electroporation. A mixture containing 100 µg/mL BIEXO, 100 µg/mL IPA, and 25 mM trehalose in PBS was electroporated at 350 V, 125 µF, 400 Ω (Gene Pulser Xcell, Bio-Rad). Post-electroporation, exosomes were washed three times by ultracentrifugation to remove unincorporated IPA. Loading efficiency was determined spectrophotometrically at 276 nm, with encapsulation efficiency calculated as (amount of IPA encapsulated/total amount of IPA added) × 100%; drug loading capacity was determined as (weight of encapsulated IPA/total weight of drug-loaded exosomes) × 100%. Release kinetics were assessed using dialysis (1 kDa MWCO) with sampling at predetermined intervals.

### FRET effect

The FRET effect was assessed during the capture of PD-1^+^ T cells labeled with DiO (excitation at 484 nm/emission at 501 nm) and LLC-MSLN cells labeled with DiI (excitation at 549 nm/emission at 565 nm) by coculturing with various concentrations of BIEXO. All of the experimental conditions involved excitation at 500 nm, with emission spectra recorded from 520 nm to 700 nm.

### Prevention of T cell exhaustion by BIEXO

Primary tumor cells and TILs were co-isolated from tumors established by subcutaneous implantation of LLC-MSLN cells in mice. Tumor cells (4 × 10⁴/well) were co-cultured with autologous TILs at an E: T ratio of 10:1 in the presence of recombinant PD-L1 (0.5 µg/mL) to induce exhaustion. Concurrently, experimental groups were treated with 0.05 µg/mL of IEXO, PIEXO, or BIEXO, while a positive control group received a PD-1 blocking antibody (0.5 µg/mL). Following a 4-h incubation, T-cell-mediated cytotoxicity was quantified using an LDH release assay according to the manufacturer’s protocol. To control for any direct cytotoxicity from the nanovesicles, the amount of LDH released from primary tumor cells incubated with each nanovesicle type alone (in the absence of TILs) was measured and subtracted from the values of the corresponding co-culture groups.

### Cytotoxicity assay

To examine the direct inhibitory effect of BIEXO on lung cancer cells, LLC-MSLN cells were co-cultured with different treatment groups (IEXO, PIEXO, MIEXO, and BIEXO) for 24 h. The cell viability was determined by CCK8 assay. To assess the cytotoxicity induced by BIEXO@IPA against lung cancer in the presence of T cells, TILs and LLC-MSLN cells (E: T = 10:1) isolated from LLC-MSLN tumor-bearing mice were co-cultured with different treatment groups (IEXO, PIEXO, BIEXO, IPA, and BIEXO@IPA) for 24 h. The apoptosis rate was determined by LDH assay.

### In vivo biodistribution of BIEXO@IPA

DiR-labeled BIEXO@IPA was obtained after co-incubating BIEXO@IPA with DiR dye for 30 min and centrifuging at 100,000 × *g* for 90 min using an ultrahigh-speed centrifuge to remove free dye. Fluorescence quantification of the major organs of the collected mice was performed *via* an IVIS imaging system at different time points (2, 4, 6, 12, 24, and 48 h) after the administration of 100 µg of DiR-labeled BIEXO@IPA *via* nebulization or tail vein injection. To investigate the dose dependence of BIEXO@IPA biodistribution in vivo, the mice were nebulized with 12.5 µg, 25 µg, 50 µg, or 100 µg of DiR-labeled BIEXO@IPA, and fluorescence quantification of major organs was performed *via* the IVIS imaging system after 12 h.

### Pharmacokinetic analysis

C57BL6/J mice received BIEXO (200 µg) or anti-PD-1/MSLN bispecific antibody (200 µg, synthesized by Creative Biolabs) *via* intravenous injection. Blood samples were collected using Multivette 600 LH-Gel tubes (SARSTEDT) at 5, 30, 60, 120, 180, 240, 360, and 480 min post-injection and centrifuged (10,000 × g, 5 min, room temperature) to obtain plasma.

For ELISA quantification, plasma samples were diluted 1:50 in PBS. BIEXO were detected using plates coated with mouse anti-His monoclonal antibody (5 µg/mL, clone HIS.H8, Thermo Fisher Scientific), followed by rabbit anti-His polyclonal antibody (2 µg/mL, Abcam, ab9108) and goat anti-rabbit IgG-HRP (BioLegend, 405306). Bispecific antibody quantification employed a dual-target sandwich ELISA. Recombinant human PD-1 protein (5 µg/mL, R&D Systems, 1086-PD) was used for capture. Bound bispecific antibodies were detected using biotinylated human CD3 protein (2 µg/mL, ACROBiosystems, CD3E-H82E9), followed by streptavidin-HRP conjugate (Thermo Fisher, Cat# N100). Signal generation was achieved with TMB substrate and absorbance measured at 450 nm. Standard curves were generated using serial dilutions of corresponding agents in PBS containing 2% mouse plasma. Plasma half-lives were determined by noncompartmental analysis using MATLAB.

### Mucus penetration studies

For mucus penetration analysis, porcine stomach mucus was utilized because of its genetic similarity and volume limitations associated with porcine tracheal tubes [62]. LLC-MSLN cells were seeded in the bottom wells of a 24-well plate (5 × 10^4^ cells per well) and allowed to adhere overnight. Subsequently, 500 µL of mucus was added to the upper wells and allowed to settle for 1 h. The DiI-labeled formulations were subsequently added to the upper wells and incubated for 6 h. After incubation, the upper wells were removed, and the cells were rinsed three times with PBS, stained with DAPI, and analyzed *via* fluorescence microscopy.

### Tumor inoculation and inhalable drug delivery

To establish the orthotopic tumor model, the mice were anesthetized with 2% isoflurane, and a 5-mm incision was made in the left chest, approximately 1 cm below the left axilla. The muscles and subcutaneous fat were carefully separated to visualize lung movement, and 5 × 10^5^ LLC-MSLN-luciferase cells suspended in 50 µL of DMEM/Matrigel were injected into the left lung tissue at a depth of 3 mm. After the injection, the incision was closed, and gentamicin and erythromycin were applied to prevent infection. The mice were monitored for 45 to 60 min until they fully recovered from anesthesia [63]. Tumor progression was quantified *via* luciferase activity *via* the intraperitoneal injection of d-luciferin bioluminescent substrate (PerkinElmer), and luminescence was measured *via* the IVIS Spectrum imaging system. Nebulized treatment commenced 6 days post tumor injection, with BIEXO@IPA, IEXO@IPA, and Lipo@IPA administered *via* a microsprayer aerosolizer (YUYAN Instrument, China) [64]. Each mouse received 117.5 µg BIEXO@IPA (17.5 µg IPA loaded in 100 µg of BIEXO). For the distribution study, the animals were euthanized 24 h after treatment, followed by analysis of BIEXO@IPA^+^, IEXO@IPA^+^, and Lipo@IPA^+^ cells.

### Therapeutic evaluation of BIEXO@IPA in lung cancer models

We evaluated the antitumor efficacy of BIEXO@IPA in three complementary lung cancer models: subcutaneous, orthotopic, and metastatic. In the subcutaneous model, 5 × 10⁵ LLC-MSLN cells were injected into the left inguinal region of C57BL/6 mice. Orthotopic lung cancer models were established as described above. Successful tumor engraftment was confirmed by bioluminescence imaging using the IVIS Spectrum system. The metastatic model was established by intravenous injection of 5 × 10⁵ B16F10-MSLN cells into the tail vein to establish pulmonary metastases. For all models, treatment groups included PIEXO (100 µg), MIEXO (100 µg), BIEXO (100 µg), IPA (17.5 µg), BIEXO@IPA (117.5 µg), and PBS as control. Therapeutic interventions were administered on days 7, 10, and 13 post-inoculation. Tumor progression was monitored using model-specific approaches: subcutaneous tumor volumes were measured with Vernier calipers, orthotopic lung tumors were assessed by bioluminescence imaging with D-luciferin (150 mg/kg, intraperitoneal injection) on days 7, 13, and 19, and metastatic burden was evaluated through nodule count, cancer cell enumeration, and lung tissue weight assessment on day 19.

### Flow cytometry analysis

Orthotopic tumors and lung tissues were harvested and immediately processed in ice-cold PBS containing 2% FBS. Tissues were mechanically dissected into 1 mm³ fragments and enzymatically digested with collagenase IV (200 U/mL, Worthington, LS004189) and DNase I (100 µg/mL, Invitrogen) for 30–60 min at 37 °C. Single-cell suspensions were obtained by filtering through 70-µm strainers.

For immune microenvironment analysis, single-cell suspensions (1 × 10⁶ cells) were blocked with anti-CD16/32 antibody (101320, BioLegend) and stained with following markers: CD8⁺ T cells (CD45⁺CD3⁺CD8⁺) using CD45-PE/Cy7 (103114, BioLegend), CD3-APC (100312, BioLegend), and CD8-PE (100708, BioLegend) combined with functional markers IFN-γ-FITC (505806, BioLegend), TNF-α-FITC (506304, BioLegend), or Granzyme B-FITC (396403, BioLegend); memory T cell subsets (CD3⁺CD8⁺CD44⁺CD62L⁺/⁻) using CD3-APC (100312, BioLegend), CD8-PE (100708, BioLegend), CD44-FITC (103005, BioLegend), and CD62L-PE/Cy7 (104417, BioLegend) to distinguish central memory (CD44⁺CD62L⁺) from effector memory (CD44⁺CD62L⁻) populations; NK cells (CD45⁺CD3⁻NK1.1⁺) using CD45-PE/Cy7 (103114, BioLegend), CD3-APC (100312, BioLegend), NK1.1-PE (108707, BioLegend), and functional markers IFN-γ-FITC (505806, BioLegend) or TNF-α-FITC (506304, BioLegend); dendritic cells (CD45⁺CD3⁻CD11c⁺MHC II⁺) using CD45-PE/Cy7 (103114, BioLegend), CD3-APC (100312, BioLegend), CD11c-PE (117307, BioLegend), and MHC II-FITC (107605, BioLegend); macrophages (CD45⁺CD3⁻F4/80⁺CD11b⁺) using CD45-PE/Cy7 (103114, BioLegend), CD3-APC (100312, BioLegend), F4/80-PE (123109, BioLegend), and CD11b-Pacific Blue (101207, BioLegend); exhausted precursor T cells (CD45⁺CD3⁺CD8⁺PD-1⁺TCF-1⁺) using CD45-PC/Cy7 (103114, BioLegend), CD3-PB450 (100227, BioLegend), CD8-PE (100708, BioLegend), PD-1-FITC (135213, BioLegend), and TCF-1-APC (566693, BD Biosciences).

For nanocarrier distribution analysis, BIEXO@IPA, IEXO@IPA, and Lipo@IPA were pre-labeled with DiD fluorescent dye prior to administration. LLC tumor cells were identified using luciferase-PE antibody (NB600-307PE, Novus). Additional tissue-resident cell populations were characterized as follows: epithelial cells (CD45⁻CD31⁻EpCAM⁺) using CD45-PE/Cy7 (103114, BioLegend), CD31-PE (561073, BD Biosciences), and EpCAM-PerCP5.5 (118219, BioLegend). DiD was used to track overall nanocarrier distribution and cellular uptake. Detailed gating strategies for identifying specific immune cell subsets and assessing nanocarrier cellular uptake are presented in Supplementary Figure S17 and Figure S18, respectively.

### Immune Cell Depletion Assay

Mice were subcutaneously inoculated in the left inguinal region with 5 × 10⁵ LLC-MSLN cells and randomly assigned to five groups. Four treatment groups received BIEXO@IPA therapy, with three of these groups additionally receiving depleting antibodies: anti-CD8 (400 µg/mouse, twice weekly, BioXcell), anti-CD4 (200 µg/mouse, once weekly, BioXcell), or anti-NK1.1 (to deplete NK cells) antibodies, respectively. The fourth treatment group received BIEXO@IPA therapy without antibody depletion. The fifth group served as a control and received PBS only.

Depleting antibodies were administered intraperitoneally beginning one day prior to BIEXO@IPA therapy and continued throughout the treatment period. BIEXO@IPA therapy was administered on days 5, 8, 11, and 14 post-tumor inoculation. Tumor growth was monitored every other day using digital calipers. In accordance with institutional animal care guidelines, mice were humanely euthanized when tumor volume reached 2,000 mm³.

### Biocompatibility experiment of BIEXO@IPA

The comprehensive biocompatibility of BIEXO@IPA was evaluated through multiple in vivo and in vitro assessments. For the in vivo safety evaluation, C57BL/6J mice received inhalation of PBS, PIEXO (100 µg), MIEXO (100 µg), BIEXO (100 µg), IPA (17.5 µg), BIEXO@IPA (117.5 µg) at three-day intervals (three total administrations). Mouse body mass was monitored biweekly as a primary physiological indicator. Systemic toxicity was assessed through comprehensive serum biochemistry, and key metabolic markers including alkaline phosphatase (ALP), alanine aminotransferase (ALT), creatinine (Cr), and blood urea nitrogen (BUN) were quantified. We conducted detailed investigations on multiple normal human cell lines to comprehensively evaluate potential cellular toxicity. Human L02 liver cells, BEAS-2B lung epithelial cells, and HK2 renal proximal tubule cells were exposed to a gradient of BIEXO@IPA concentrations (0–100 µg/mL) for 24 h. Cell viability was quantified *via* a CCK8 assay, with the absorbance measured at 450 nm, and viability was calculated relative to that of the control groups. Potential tumorigenicity, including AFP, CEA, MUC1, CA125, and CA19-9, was further investigated by analyzing serum tumor marker profiles in C57BL6/J mice. Critical organs (heart, liver, spleen, lungs, and kidneys) were subjected to comprehensive histological examination to detect any potential structural or functional anomalies.

### Evaluation of BIEXO@IPA for potentiating CAR-T cell antitumor activity in vivo

To determine whether BIEXO@IPA augments CAR-T efficacy, we employed two complementary tumor models. For the orthotopic model, C57BL/6J mice were inoculated intratracheally with LLC-MSLN-luciferase cells. For the metastatic model, mice received 2 × 10^5^ B16F10-MSLN cells via tail vein injection.

Following tumor establishment (day 7 for orthotopic, day 3 for metastatic model), mice were randomized into four treatment groups: (1) PBS control; (2) 1 × 10^6^ MSLN CAR-T cells (i.v.); (3) 1 × 10^6^ CAR-T cells (i.v.) plus 117.5 µg anti-PD-1 antibody (MCE, HY-P990824) (inh.); or (4) 1 × 10^6^ CAR-T cells (i.v.) plus 117.5 µg BIEXO@IPA (inh.).

For the orthotopic model, tumor burden was monitored longitudinally via bioluminescence imaging (IVIS) after D-luciferin administration (150 mg/kg, i.p.) on days 7, 14, and 21. Survival was recorded daily until day 80. In the metastatic model, lungs were harvested on day 14 for enumeration of surface nodules, weight measurement, and flow cytometric quantification of B16F10-MSLN cells.

For the humanized model, peripheral blood and tumor biopsy samples were obtained from the lung cancer patient. Human samples were collected with written informed consent under Medical Ethics Committee approval (#K140-1). PBMCs were isolated using Ficoll density gradient centrifugation and used for CAR-T cell generation. NSG mice were inoculated with the primary tumor cells (MSLN-Luciferase-GFP) into the left lung parenchyma. On day 7 post-inoculation, mice received an intravenous injection of human MSLN-CAR-T cells (5 × 10⁶), concurrently with nebulized inhalation of BIEXO@IPA (117.5 µg per mouse) on days 7, 10, and 13. Tumor burden was monitored using IVIS imaging on days 7, 13, and 19. Survival was recorded daily until day 40.

### Tumor rechallenge and memory T cell analysis

To evaluate immune memory formation, C57BL/6 mice were initially inoculated subcutaneously with 2 × 10^5^ LLC-MSLN-luciferase cells in the left inguinal region. Following tumor engraftment confirmation, mice received BIEXO@IPA + CAR-T treatment on days 7, 10, and 13 post-inoculation. Mice achieving complete remission were rechallenged on day 110 with 2 × 10^5^ LLC-MSLN-luciferase cells, with naïve mice serving as controls. Tumor growth was monitored every other day using IVIS imaging following D-luciferin injection (10 mg/kg).

For memory T cell characterization, tumor-eradicated mice received LLC-MSLN-luciferase cell lysates (10 µg per mouse) on day 120 after primary inoculation. PBMCs were collected and analyzed by flow cytometry. Central memory CD8^+^ T cells were identified as CD8^+^CD44^+^CD62L^+^, while effector memory T cells were defined as CD8^+^CD44^+^CD62L^−^.

### Statistical analysis

All data are presented as mean ± standard deviation (SD). Sample size (n) represents the number of biological replicates, as specified in the figure legends. Normality of data distribution was assessed using the Shapiro-Wilk test. For comparisons between two groups, two-tailed unpaired Student’s t-tests were used. For comparisons among more than two groups, one-way analysis of variance (ANOVA) followed by Tukey’s multiple comparisons test was performed. For comparisons involving two independent variables (e.g., tumor growth curves, cytotoxicity assays, and pharmacokinetic profiles), two-way analysis of variance (ANOVA) followed by Tukey’s multiple comparisons test was performed. Survival data were analyzed using the Kaplan-Meier method and compared using the Log-rank (Mantel-Cox) test. GraphPad Prism 8 was used for all statistical analysis, and the following levels of statistical significance were applied: *p < 0.05, **p < 0.01, ***p < 0.001, and ****p < 0.0001; ns, not significant.

## Supplementary Information

Supplementary Material 1.

## Data Availability

All data supporting the findings of this study are available from the corresponding author upon reasonable request.
