# Supplementary material for: Engineering BiTE-inspired IPSC-exosomes to potentiate CAR-T cell therapy against lung cancer
Source: J Nanobiotechnology. 2026 Mar 17;24:388. doi: 10.1186/s12951-026-04242-3 (PMC13107855; doi:10.1186/s12951-026-04242-3)
Supplement: Supplementary file 1 — Supplementary Material 1. [file 12951_2026_4242_MOESM1_ESM.docx]

### Supplementary Material

**Engineering BiTE-Inspired IPSC-Exosomes to Potentiate CAR-T Cell Therapy Against Lung Cancer**

Ronghao Wang^1†^, Guining Fu^1†^, Haozhao Dou^1†^, Mingyuan Hu^1†^, Zhanglin Li^1†^, Junwen Gan^1^, Jiasheng He^1^, Xiaojian Li^1^, Guihong Zhang^1^, Xianjun Li^3^*, Tianchuan Zhu^2^ *, Qingdong Cao^1^*


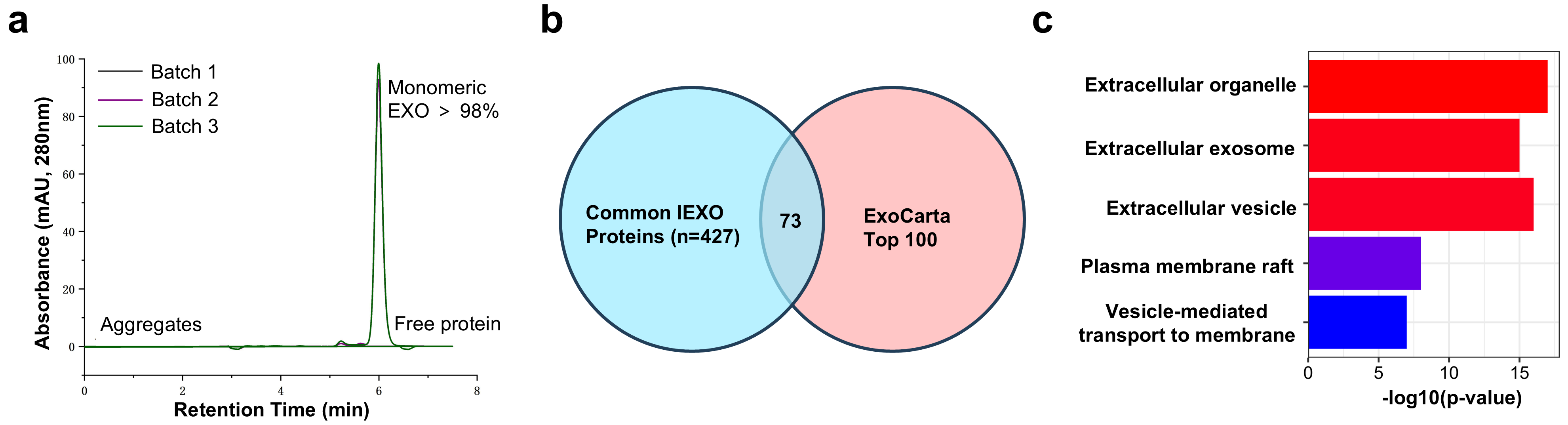


**Figure S1. Quality control and proteomic validation of iPSC-derived exosomes.** (a) Representative SEC-HPLC chromatogram of IEXOs eluted from a qEV column. The single peak at the void volume indicates the isolation of intact vesicles separated from free protein fractions. (b) Venn diagram depicting the proteomic identity of IEXOs. The blue circle represents the consensus high-abundance proteome derived from the intersection of the top 500 proteins identified in each of three independent batches. The red circle represents the top 100 exosome markers from the ExoCarta database. The overlap region indicates that canonical exosomal markers are consistently enriched in the IEXO formulation. (c) Gene Ontology (GO) cellular component enrichment analysis of the top 500 consensus proteins shared across three independent batches.


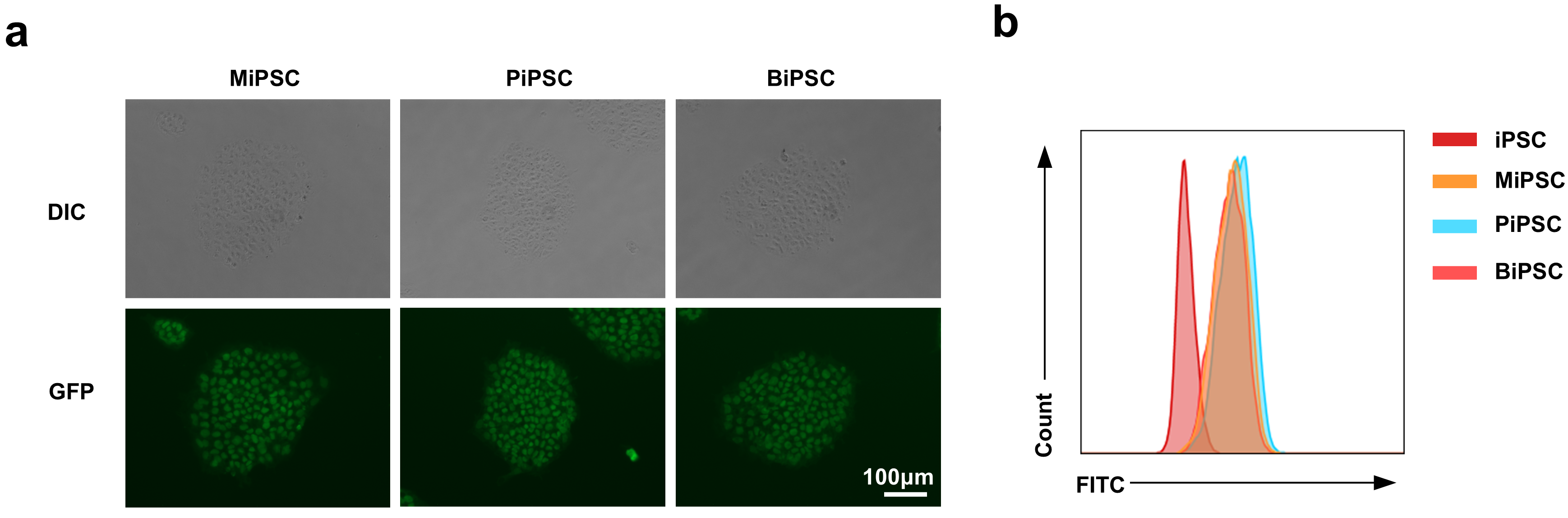


**Figure S2. Validation of scFv construct expression in engineered iPSC.** (a) Fluorescence microscopy images of mouse induced pluripotent stem cells (iPSCs) following lentiviral transduction with constructs expressing anti-MSLN scFv, anti-PD-1 scFv, or bispecific PD-1/MSLN scFv. Cells were fixed with 0.4% paraformaldehyde (PFA) prior to imaging. (b) Flow cytometric analysis of surface expression of the corresponding scFv on transduced iPSCs.


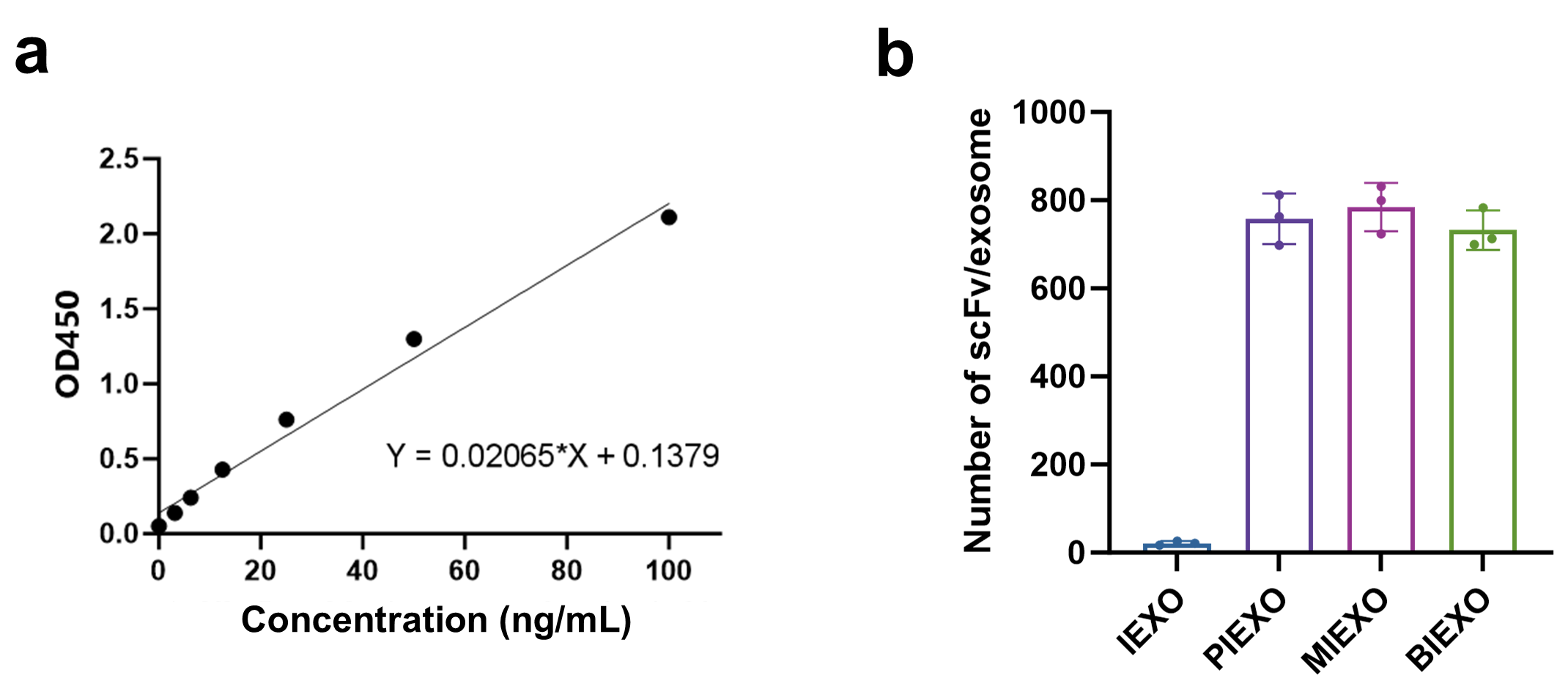


**Figure S3. Quantification of scFv display on engineered exosomes.** (a) ELISA standard curve using a recombinant 6×His-tagged protein standard. (b) Number of scFv molecules per exosome for PIEXO, MIEXO, and BIEXO (n = 3).


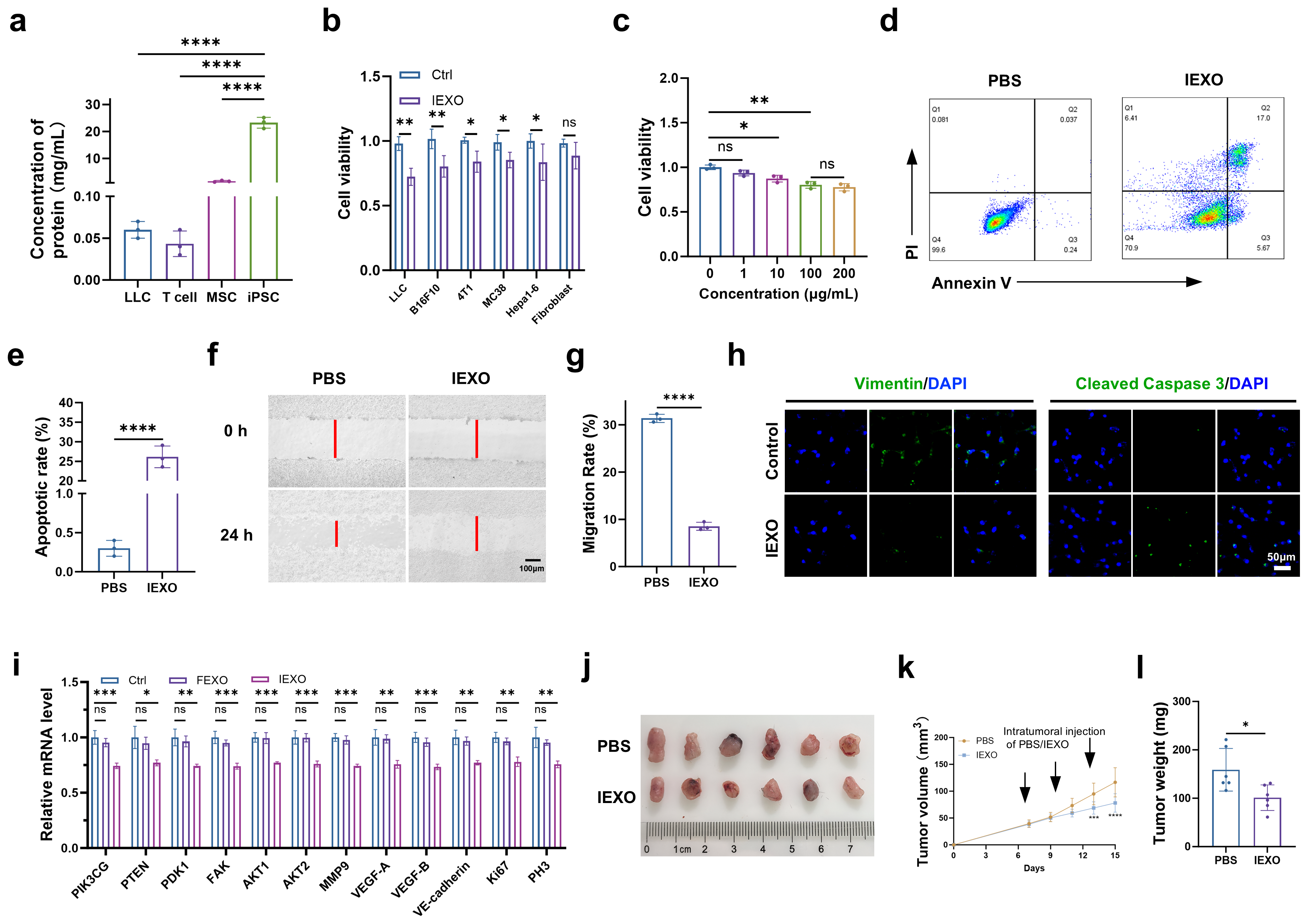
**Figure S4. Characterization and antitumor properties of IEXOs.** (a) Exosomal protein yield from iPSCs compared to LLC cells, T cells, and MSCs (n = 3). (b) Assessment of IEXO (100 μg/mL)-mediated growth inhibition across various tumor cell lines after co-incubated for 24 h (n = 3). (c) Dose-dependent inhibition of LLC cell proliferation by IEXOs after co-incubated for 24 h (n = 3). (d, e) Apoptosis analysis by Annexin V/PI staining with representative plots (d) and quantification (e) (n = 3). (f, g) Wound-healing assay showing IEXO-mediated suppression of LLC cell migration with representative images (f) and quantification (g) (n = 3). (h) Immunofluorescence analysis of vimentin and cleaved caspase-3 expression in LLC cells following IEXO treatment. (i) Quantitative RT-PCR analysis of proliferation- and migration-related genes in LLC cells treated with IEXO, using fibroblast-derived exosomes (FEXO) as a control. (j-l) In vivo therapeutic efficacy of IEXOs in subcutaneous LLC tumor-bearing mice (n = 6), including tumor volume progression (j, k) and final tumor weight on day 15 (l). The data are presented as the mean ± SD. Statistical significance was determined via one-way ANOVA with Tukey's post-hoc test (a, c), two-way ANOVA with Tukey's post-hoc test (b, i, k), and unpaired two-tailed Student's t-test (e, g, l). **p < 0.05, **p < 0.01, ****p < 0.0001. ns, not significant.*


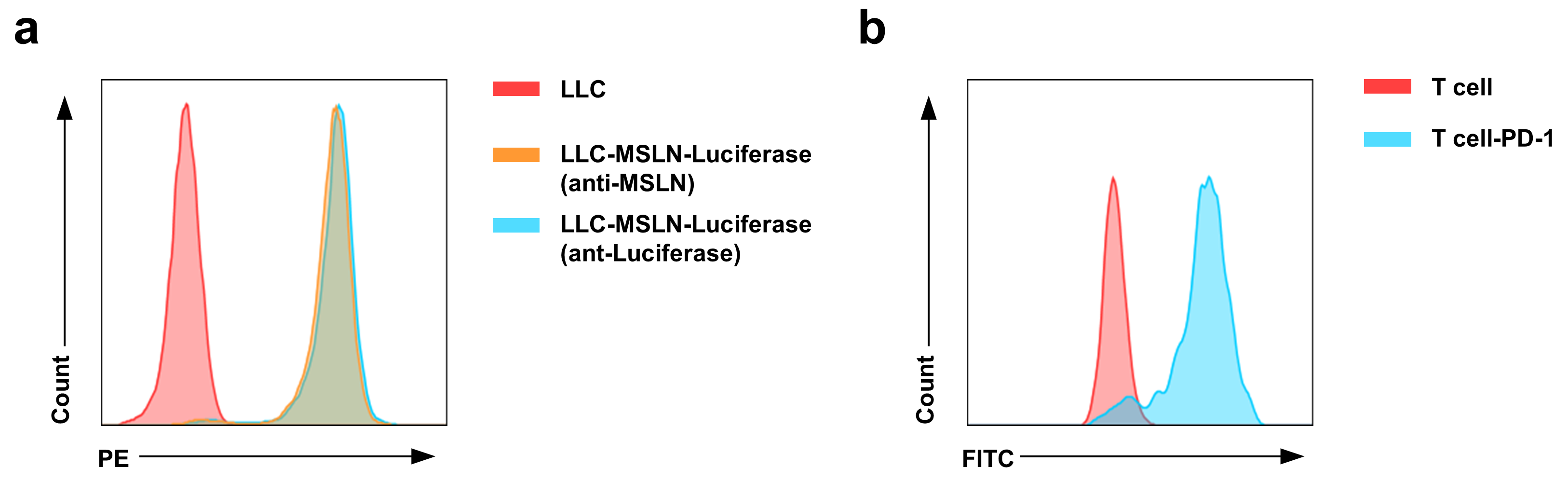


**Figure S5.** Flow cytometric validation of PD-1 and MSLN expression in different cell populations. (a) Flow cytometric analysis confirming successful expression of MSLN and Luciferase in LLC cells following lentiviral transduction. (b) Flow cytometric validation of PD-1-positive T cells isolated from mouse splenocytes by fluorescence-activated cell sorting (FACS).


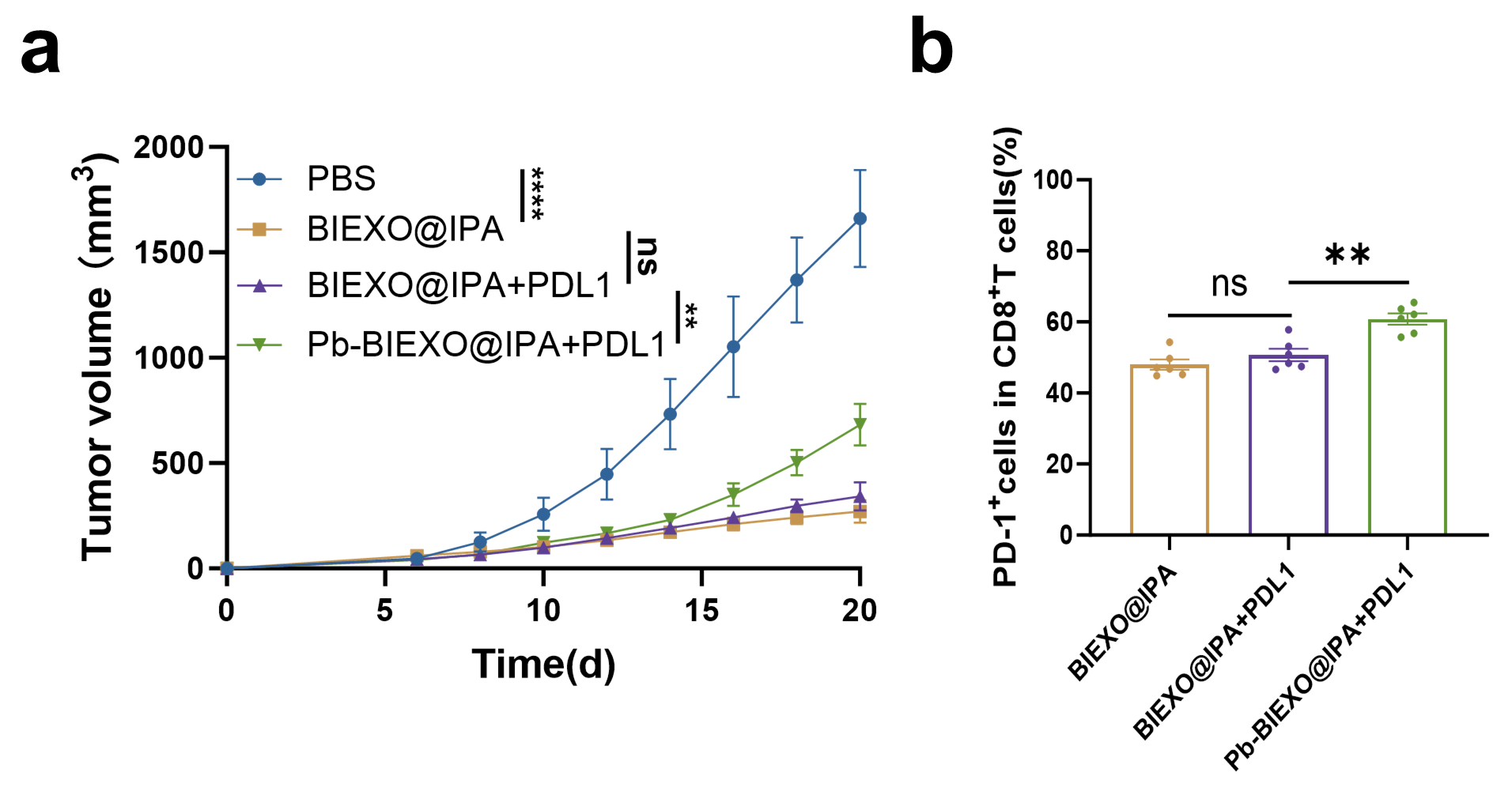


**Fig. S6. Rescue from PD-L1-mediated T-cell exhaustion by BIEXO@IPA.** a, *In vivo* assessment of PD-L1-mediated exhaustion of BIEXO@IPA, monitored through LLC-MSLN tumor growth after intratumoral injection of BIEXO@IPA with or without exogenous PD-L1, and Pb-BIEXO@IPA with exogenous PD-L1 (n = 6). b, CD8^+^ T-cell exhaustion was quantified as the percentage of PD-1^+^ cells among CD8^+^ T cells in tumor tissues on day 20 (n = 6). Data are mean ± SD. Statistical significance was determined by two-way ANOVA with Tukey's post-hoc test (a) (annotations correspond to the final day) and one-way ANOVA with Tukey's post-hoc test (b). ***p < 0.01, ****p < 0.0001, ns*, *not significant.*


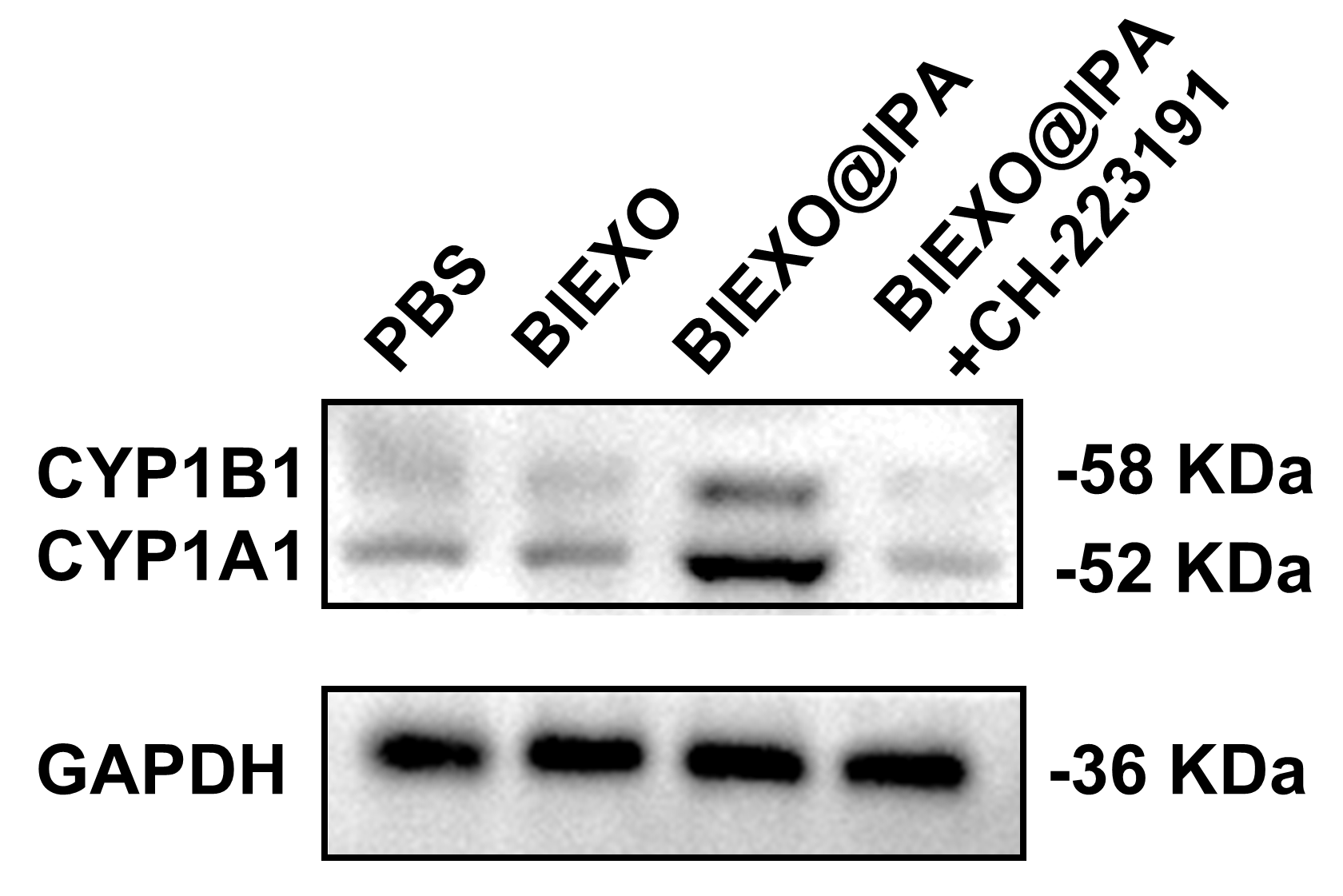


**Figure S7. BIEXO@IPA upregulates downstream targets via AhR pathway activation.** Western blot analysis of AhR downstream target proteins (CYP1A1 and CYP1B1) in CD8^+^ T cells following pre-treatment with the AhR antagonist CH-223191 and subsequent stimulation with BIEXO@IPA.


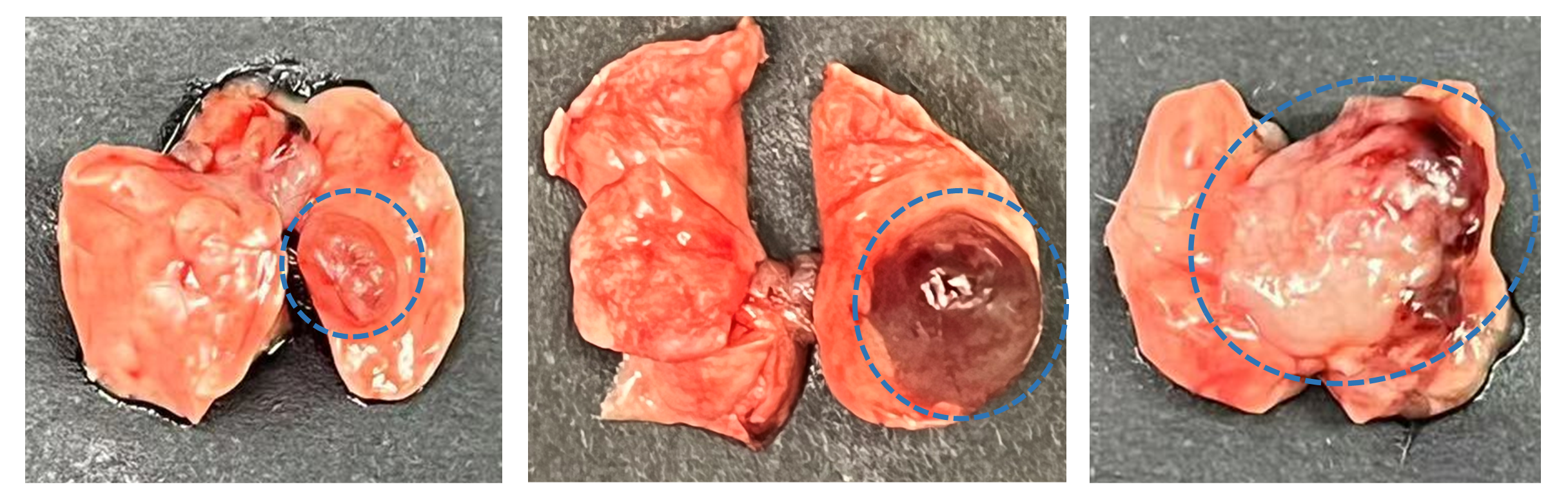


**Figure S8. Establishment of orthotopic lung cancer model.** Representative images demonstrating successful establishment of the orthotopic lung cancer model.


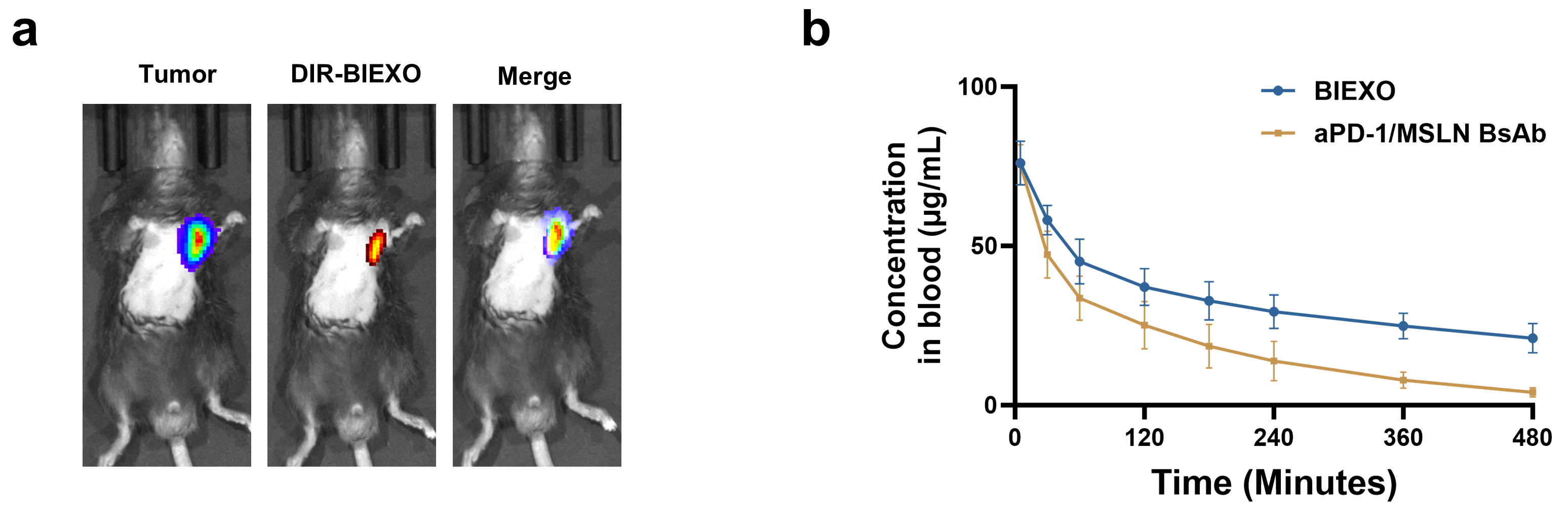


**Figure S9. *In vivo* tracking and pharmacokinetics of nebulized BIEXO@IPA in an orthotopic lung cancer model.** (a) Co-localization analysis of nebulized DIR-labeled BIEXO@IPA in an orthotopic lung tumor model established using LLC-MSLN-luciferase cells. (b) Time-dependent clearance profile of BIEXO in blood sample following nebulized administration (n = 4). The data are presented as the mean ± SD. Statistical significance was determined via two-way ANOVA with Tukey's post-hoc test for multiple comparisons (b). *****p < 0.0001.*


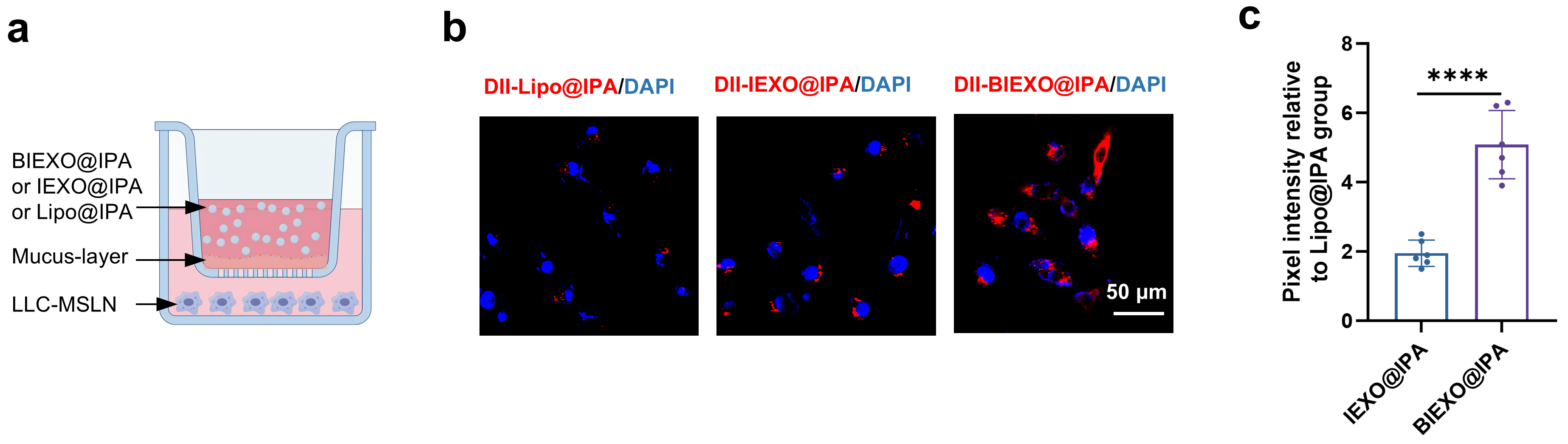


**Figure S10. Assessment of nanovesicle penetration through mucus barrier and subsequent cellular uptake.** **a,** Schematic illustration of the trans-well experimental setup used to evaluate mucus penetration and cellular uptake efficiency. **b,** Representative immunofluorescence microscopy images of LLC cells following 6-hour incubation with DiI-labeled nanovesicles (BIEXO@IPA, IEXO@IPA, or Lipo@IPA; 100 µg/mL). Cell nuclei were counterstained with DAPI. **c,** Quantitative analysis of cellular uptake efficiency for different nanovesicle formulations, expressed as percentage of DiI-positive cells relative to total cell count (n = 6). The data are presented as the mean ± SD. Statistical significance was determined via unpaired t-test **(c)**. *****p <0.0001*.


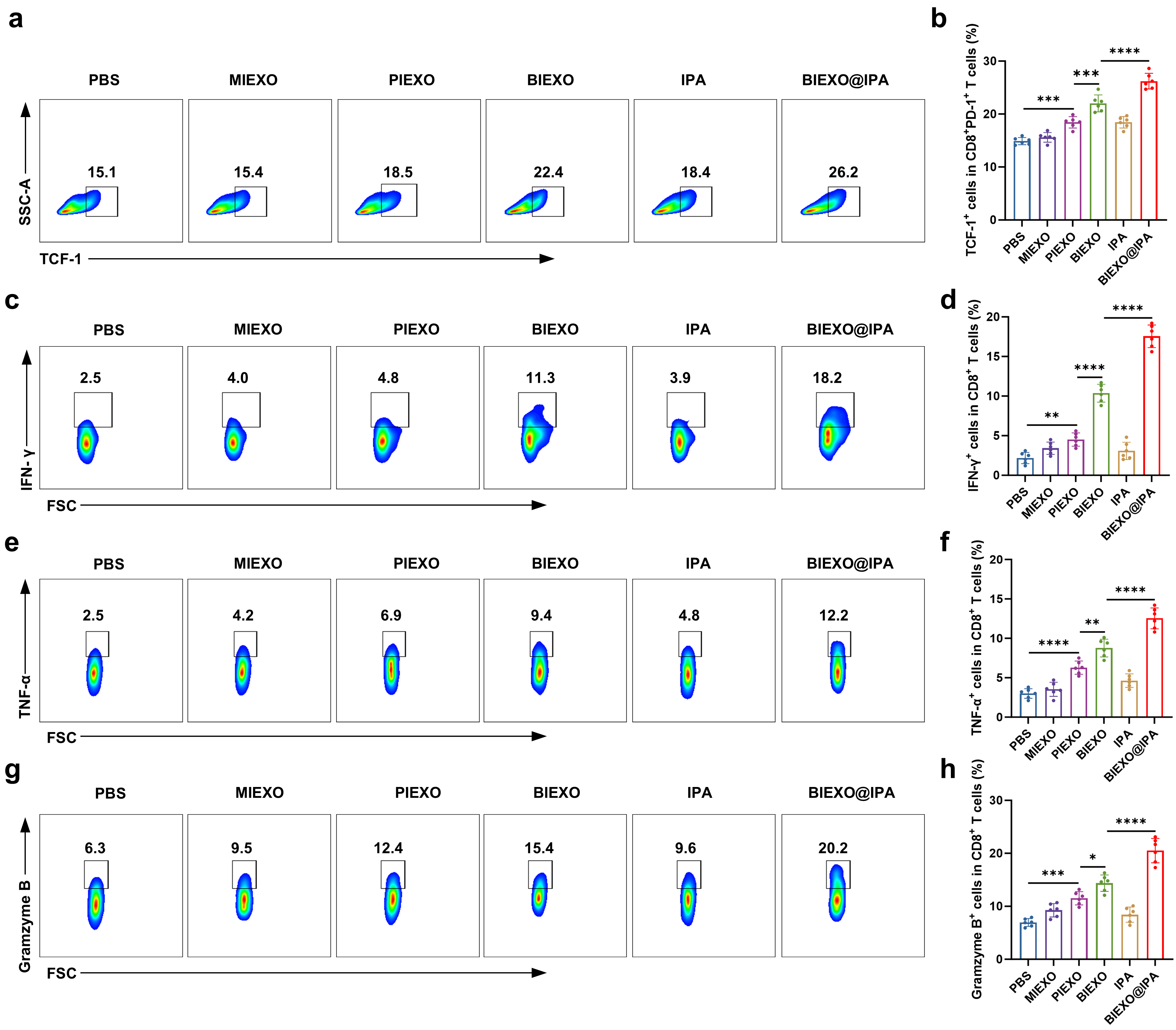


**Figure S11. Flow cytometric analysis of tumor-infiltrating immune cells in orthotopic LLC tumors.** (a, b) Representative flow cytometry plots (a) and quantitative analysis (b) of the proportion of TCF-1⁺ cells among the PD-1⁺CD8⁺ T cell population (n = 6). (c–h) Functional analysis of tumor-infiltrating CD8^+^ T cells, including IFN-γ^+^ cells (c, d), TNF-α^+^ cells (e, f), and Granzyme B^+^ cells (g, h) (n =6), via flow cytometry. Statistical significance was determined by one-way ANOVA with Tukey's post-hoc test for multiple comparisons. **p < 0.05, **p < 0.01, ***p < 0.001, ****p < 0.0001; ns, not significant.*


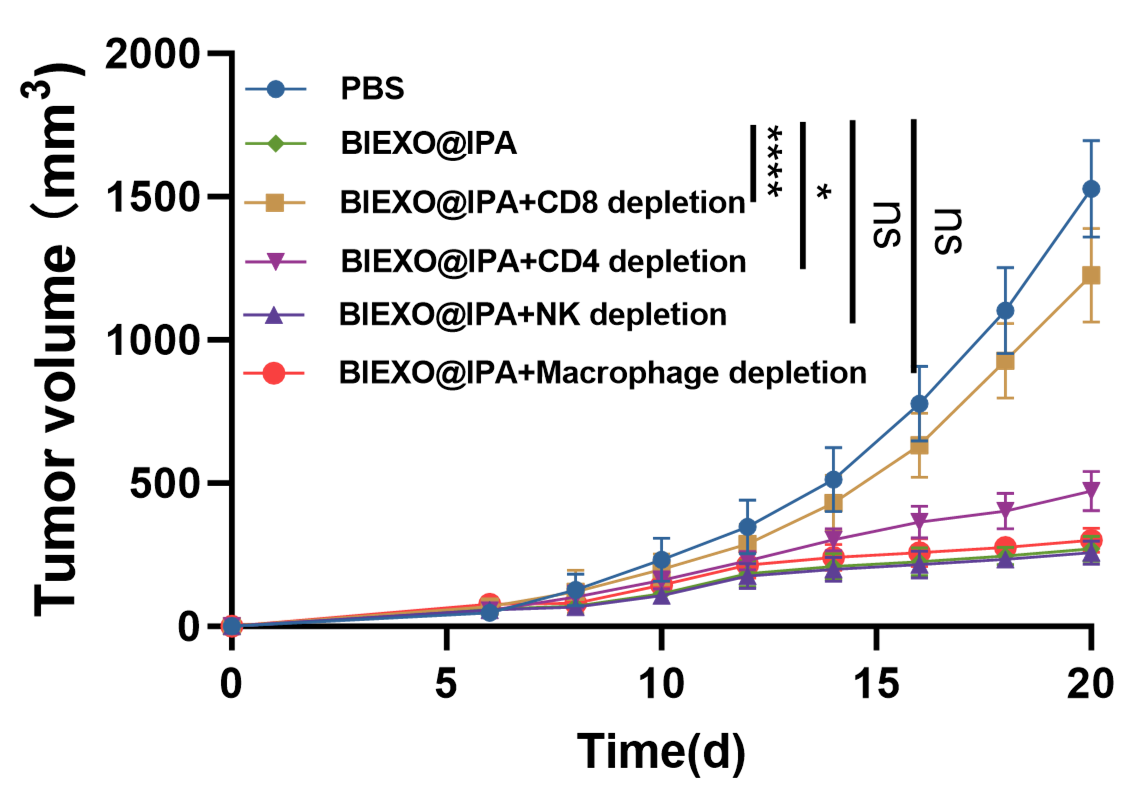


**Figure S12. Impact of immune cell depletion on BIEXO@IPA therapeutic efficacy in subcutaneous LLC tumor model.**Tumor volume (mm³) was monitored in mice receiving different treatment regimens: BIEXO@IPA alone, BIEXO@IPA with CD8^+^ T cell depletion, BIEXO@IPA with CD4^+^ T cell depletion, BIEXO@IPA with NK cell depletion, or BIEXO@IPA with macrophage depletion (n = 6). Data are presented as mean ± SD. Statistical significance was determined by two-way ANOVA followed by Tukey’s test, with annotations shown for the final day. *****p < 0.0001*; ns, not significant.


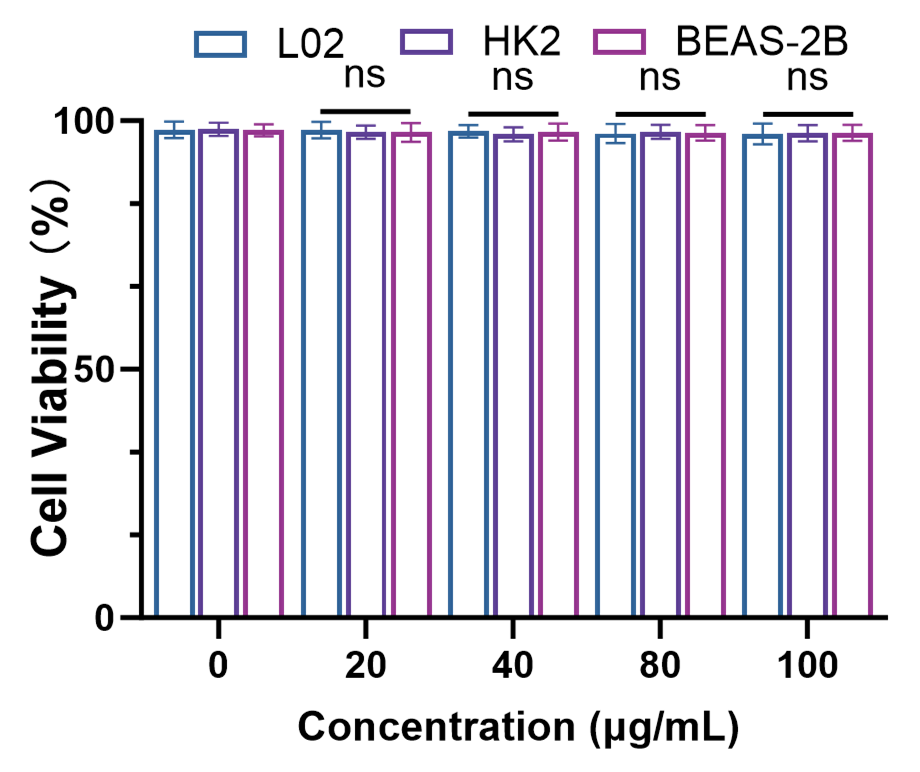


**Figure S13. In vitro cytotoxicity assessment of BIEXO@IPA in normal human cell lines.** Cell viability assessment of human L02 cells, human BEAS-2B normal lung cells, and HK2 normal kidney proximal tubule epithelial cells following BIEXO@IPA treatment. The data are presented as the mean ± SD. All treatment groups were compared against the 0 μg/mL control group for statistical analysis. *ns, not significant.*


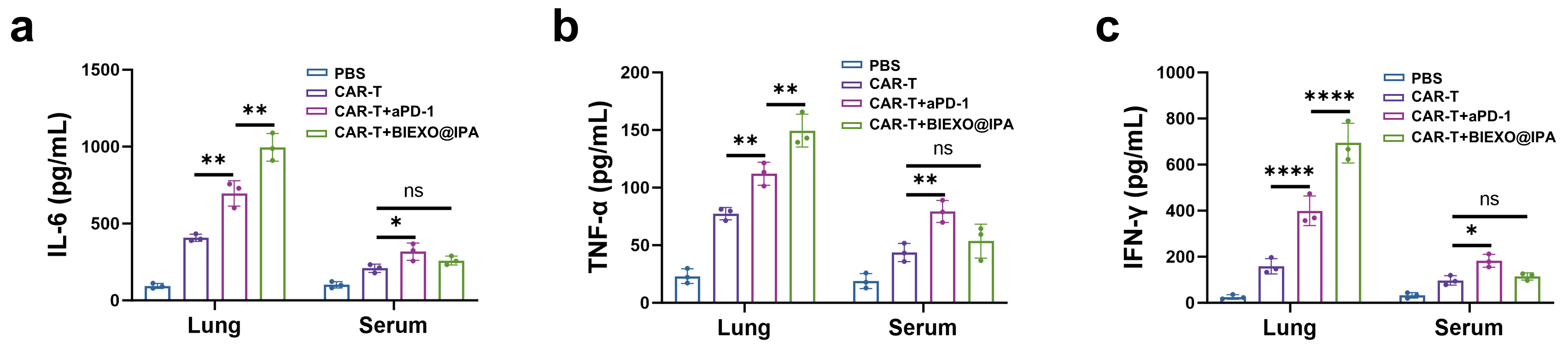


**Figure S14. Cytokine levels in bronchoalveolar lavage fluid (BALF) and serum measured by ELISA.** (a) IL-6, (b) TNF-α, and (c) IFN-γ (n = 3). Data are presented as mean ± SD. Statistical significance was determined via two-way ANOVA with multiple comparisons with Tukey's multiple comparisons test. **p < 0.05, **p < 0.01, ****p < 0.0001; ns, not significant.*


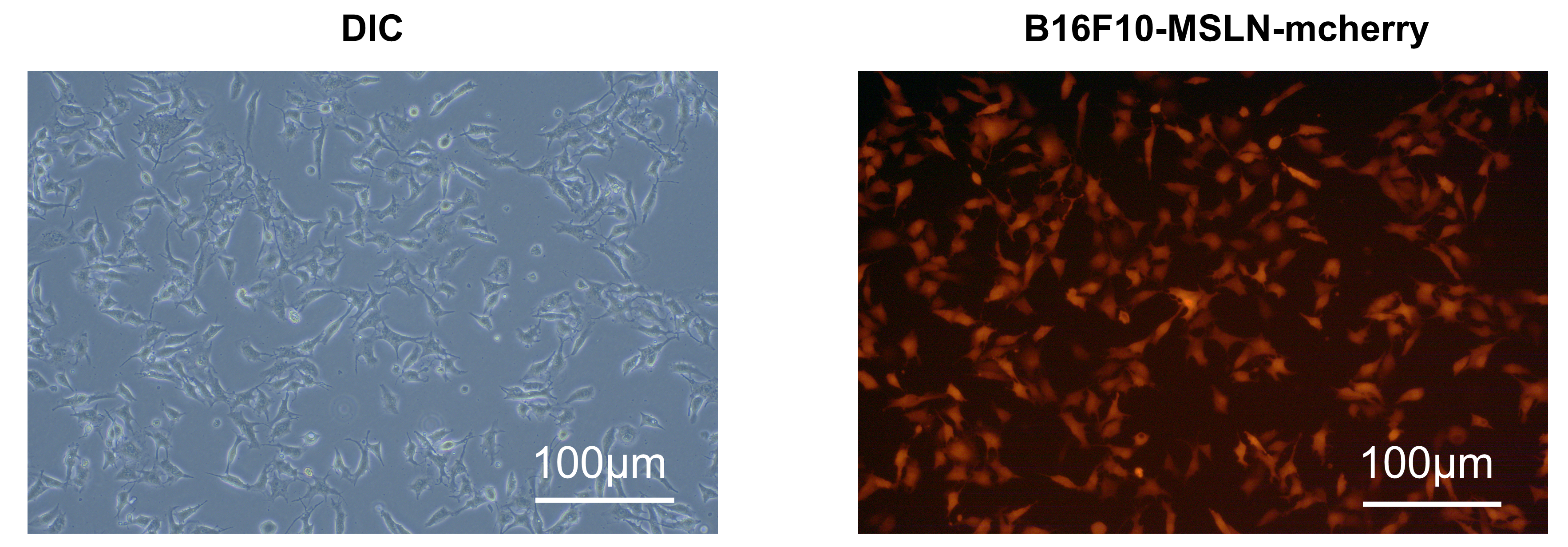


**Figure S15. Generation of B16F10-MSLN cell line.** Fluorescence microscopy analysis of mCherry expression in B16F10 cells following lentiviral transduction with MSLN-mCherry construct.


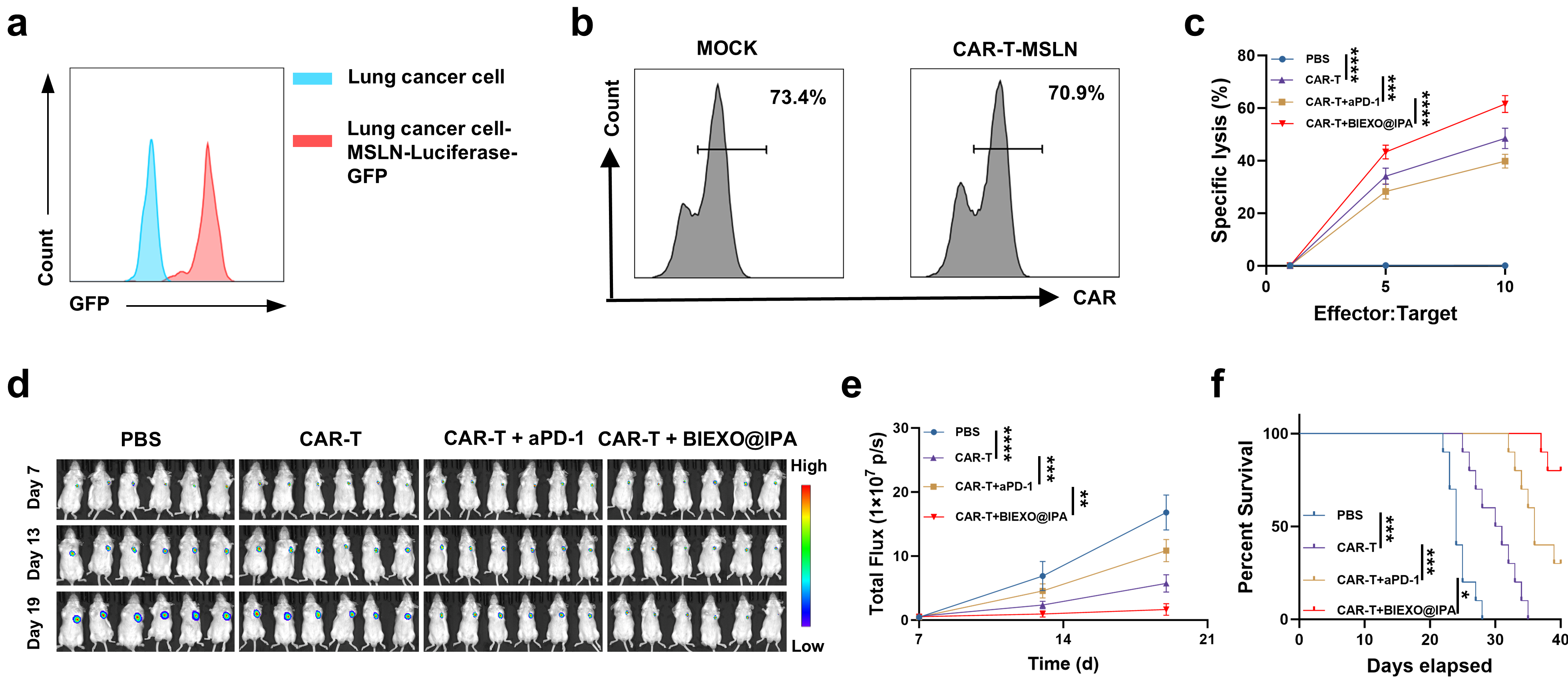


**Figure S16. Validation of efficacy in patient-derived primary lung cancer cells and orthotopic PDX models.** (a) Flow cytometric analysis confirming the stable expression of MSLN-Luciferase-GFP in primary lung cancer cells following lentiviral transduction. **(b)** Flow cytometric validation of CAR expression on T cells. **(c)** Cytotoxicity of MSLN-CAR-T cells against primary lung cancer cells determined by LDH release assay. (d, e) Longitudinal bioluminescence imaging (d) and corresponding quantification of total flux (e) in orthotopic PDX models established from MSLN-Luciferase-expressing primary lung cancer cells on days 7, 13, and 19 (n=6). (f) Kaplan-Meier survival curves (n = 10). The data are presented as the mean ± SD. Statistical significance was determined via two-way ANOVA with Tukey's post-hoc test (c, e), and log-rank test (f). Significance is annotated at the 10:1 E:T ratio for (c) and at the endpoint for the tumor growth curve in (e). **p < 0.05, **p < 0.01, ****p < 0.0001. ns, not significant.*


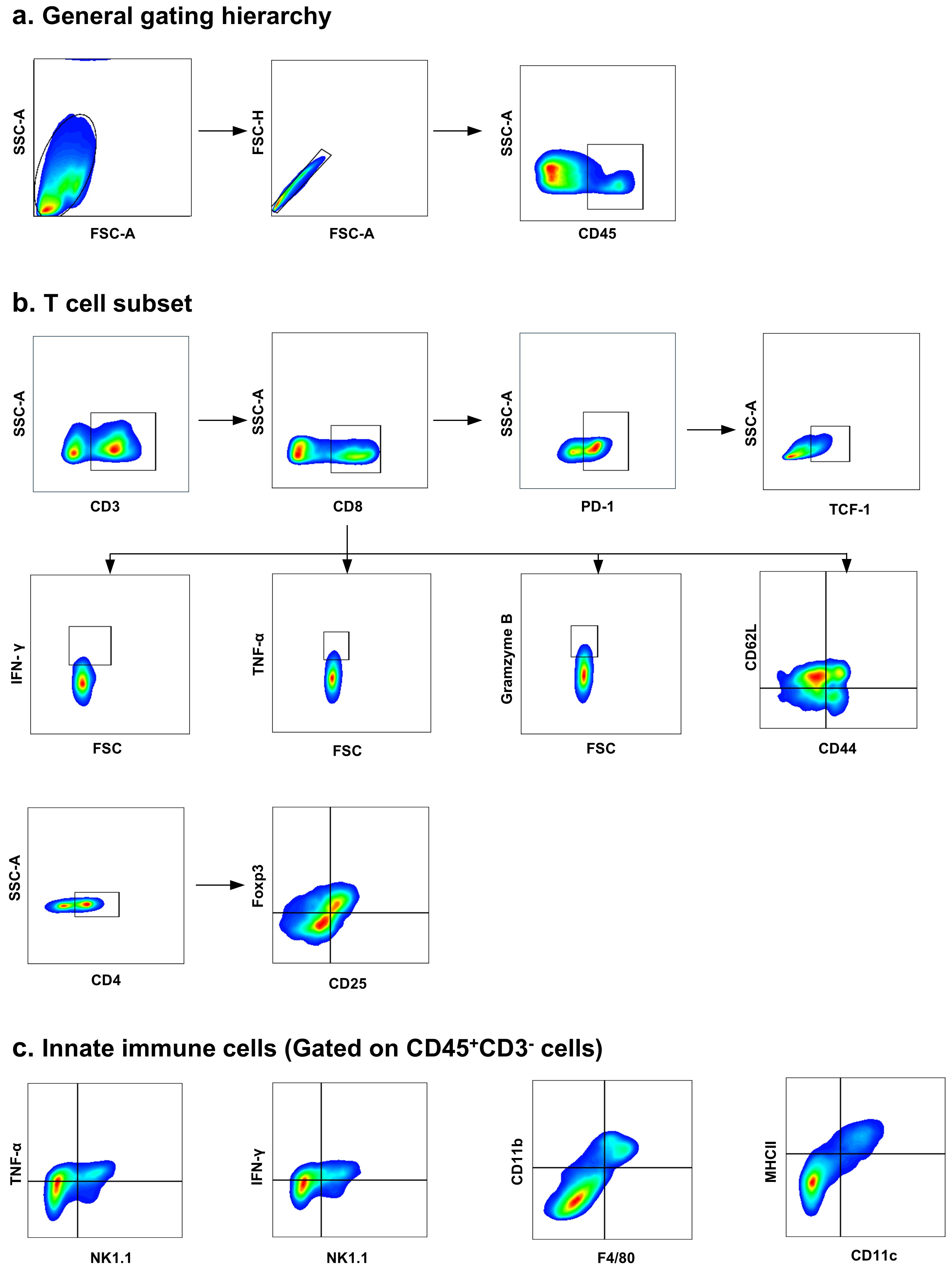


**Figure S17. Flow cytometry gating strategy for immune profiling of the tumor microenvironment.** C57BL/6 mice bearing LLC tumors were treated with PBS, MIEXO, PIEXO, BIEXO, IPA, or BIEXO@IPA. Single-cell suspensions from tumor tissues were stained with antibodies against lineage and functional markers. Representative plots display the hierarchical gating strategies used to identify CD4⁺ T cells, regulatory T cells (Tregs), CD8⁺ T cells (including IFN-γ⁺, TNF-α⁺, Granzyme B⁺ subsets, and Tpex cells), Macrophages, DCs, and NK cells (including IFN-γ⁺, TNF-α⁺ subsets). These gating strategies correspond to the quantitative data presented in Figure 4f–g, j–r and Figure S11.


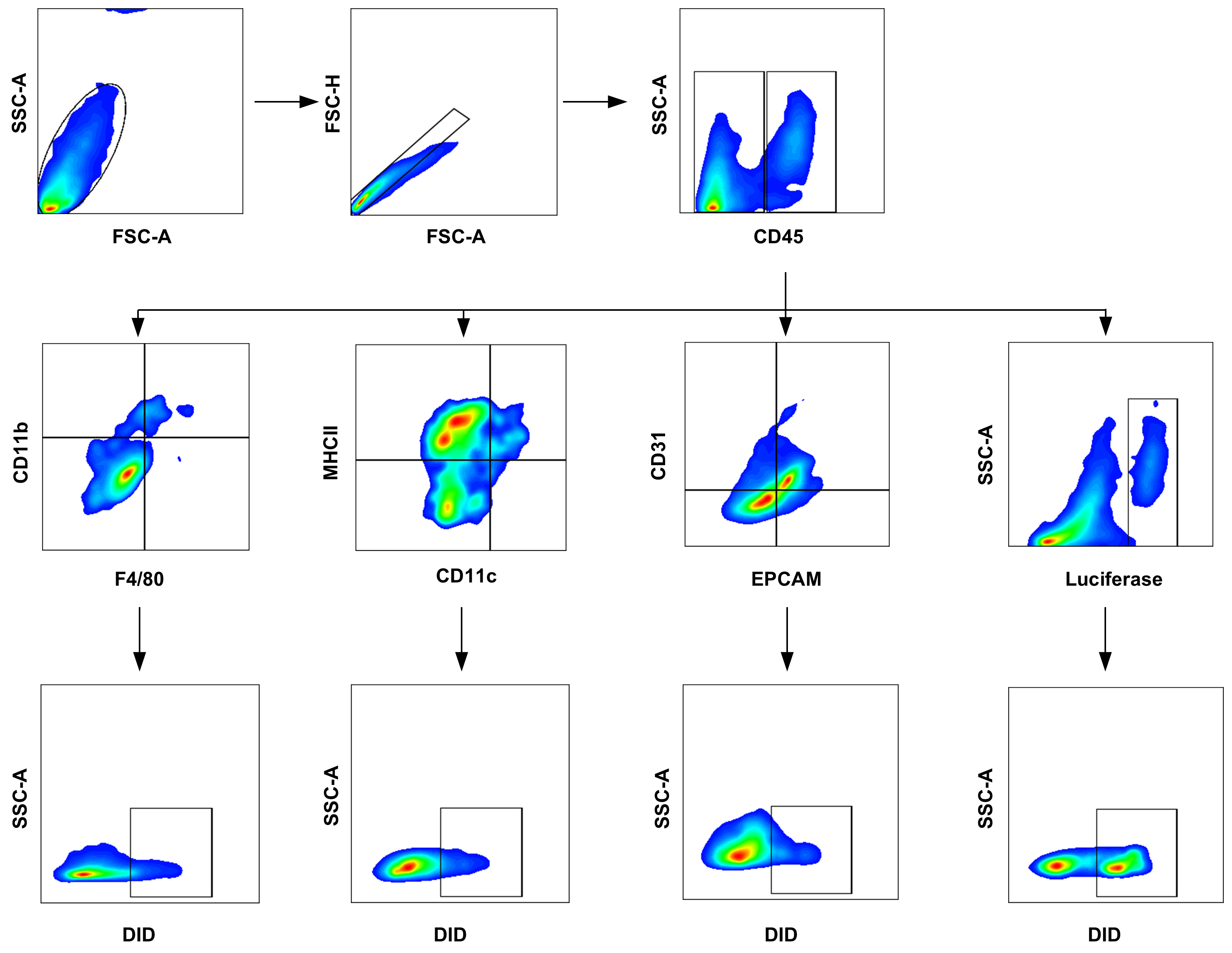


**Figure S18. Flow cytometry gating strategy for analyzing nanocarrier biodistribution in lung tissue.** C57BL/6 mice bearing LLC tumors were treated with BIEXO@IPA, IEXO@IPA, or Lipo@IPA. Lung single-cell suspensions were stained with antibodies against specific cell lineage markers, followed by flow cytometry analysis. Representative plots display the hierarchical gating strategy used to identify LLC-Luciferase tumor cells, epithelial cells, dendritic cells (DCs), and macrophages for figures 3k-n.

**Table S1. Batch-to-batch consistency analysis of iPSC-derived exosomes**

| **Parameter** | **Batch 1** | **Batch 2** | **Batch 3** | **CV (%)** |
| --- | --- | --- | --- | --- |
| Particle size (nm) | 143.3 | 147.7 | 145.6 | 3.2 |
| Zeta potential (mV) | -24.6 | -26.1 | -23.8 | 4.7 |
| Protein concentration (mg/mL) | 20.5 | 19.8 | 19.2 | 3.3 |
| Particle concentration (particles/mL) | 2.3 × 10^11^ | 1.9 × 10^11^ | 2.1 ×10^11^ | 9.5 |

Note: CV, coefficient of variation. Particle size and concentration were measured by nanoparticle tracking analysis. Zeta potential was determined by dynamic light scattering. Protein concentration was quantified by BCA assay.
